# Effects of Internet-Based Cognitive Behavioral Therapy in Routine Care for Adults in Treatment for Depression and Anxiety: Systematic Review and Meta-Analysis

**DOI:** 10.2196/18100

**Published:** 2020-08-31

**Authors:** Anne Etzelmueller, Christiaan Vis, Eirini Karyotaki, Harald Baumeister, Nickolai Titov, Matthias Berking, Pim Cuijpers, Heleen Riper, David Daniel Ebert

**Affiliations:** 1 Department of Clinical Psychology and Psychotherapy Friedrich-Alexander University Erlangen-Nuremberg Erlangen Germany; 2 GET.ON Institute GmbH Hamburg Germany; 3 Department of Clinical, Neuro-, & Developmental Psychology Faculty of Behavioural and Movement Sciences VU Amsterdam Amsterdam Netherlands; 4 Amsterdam Public Health Research Institute Mental Health Amsterdam Netherlands; 5 Department of Global Health and Social Medicine Harvard Medical School, USA Boston, MA United States; 6 Department of Clinical Psychology and Psychotherapy Institute of Psychology and Education, Ulm University Ulm Germany; 7 eCentre Clinic Department of Psychology Macquarie University Sydney Australia; 8 Community Mental Health Centre GGZ inGeest Amsterdam Netherlands

**Keywords:** internet-based interventions, depression, anxiety, effectiveness, acceptability, routine care

## Abstract

**Background:**

Although there is evidence for the efficacy of internet-based cognitive behavioral therapy (iCBT), the generalizability of results to routine care is limited.

**Objective:**

This study systematically reviews effectiveness studies of guided iCBT interventions for the treatment of depression or anxiety.

**Methods:**

The acceptability (uptake, participants’ characteristics, adherence, and satisfaction), effectiveness, and negative effects (deterioration) of nonrandomized pre-post designs conducted under routine care conditions were synthesized using systematic review and meta-analytic approaches.

**Results:**

A total of 19 studies including 30 groups were included in the analysis. Despite high heterogeneity, individual effect sizes of investigated studies indicate clinically relevant changes, with effect sizes ranging from Hedges’ g=0.42-1.88, with a pooled effect of 1.78 for depression and 0.94 for anxiety studies. Uptake, participants’ characteristics, adherence, and satisfaction indicate a moderate to high acceptability of the interventions. The average deterioration across studies was 2.9%.

**Conclusions:**

This study provides evidence supporting the acceptability and effectiveness of guided iCBT for the treatment of depression and anxiety in routine care. Given the high heterogeneity between interventions and contexts, health care providers should select interventions that have been proven in randomized controlled clinical trials. The successful application of iCBT may be an effective way of increasing health care in multiple contexts.

## Introduction

Depressive and anxiety disorders are common mental health problems associated with significant suffering, impairment, and reduction in the quality of life [1,2]. Both disorders lead to considerable socioeconomic costs through decreased work productivity and higher utilization of health care services [3,4].

Despite the proven effectiveness of psychotherapy in the treatment of depression and anxiety [5], the provision of evidence-based treatments depicts a constant challenge given the barriers such as the shortage of treatment, uneven distribution of trained providers, delayed treatment provision, and inadequacy of treatment [6,7]. Furthermore, research on patients’ preferences has shown that many do neither make use of psychotherapeutic treatments nor do they receive psychopharmacological treatment [7]. Using the internet to provide psychotherapeutic interventions may increase the coverage of usual care services [8,9] by providing highly accessible and scalable interventions reaching people who cannot be reached otherwise. Recent research suggests that internet-based cognitive behavioral therapy (iCBT) with therapeutic guidance is effective for the prevention [10,11] and treatment [12-15] of common mental disorders. Systematic reviews on studies were also able to show comparable effects to face-to-face treatments in adults [16,17]. In a recent meta-analysis, Romijn et al [13] showed that iCBT interventions for anxiety disorders can also have significant effects obtained in trials implemented in clinical care. They also found that effects were smaller in samples recruited in clinical practice than in samples recruited with an open recruitment method compared with waitlist-control groups [13], which raises the question of the effects of iCBT when implemented in routine practice.

Although randomized controlled trials (RCTs) are considered the gold standard in exploring the efficacy of mental health interventions, the idealized and controlled nature of these trials limits the generalizability of findings to routine care populations [18]. RCTs maximize the internal validity, to ensure that the effect found can be attributed to the investigated intervention [19,20]. Thus, RCT findings are restricted by controlled protocols, explicit eligibility criteria, and patient recruitment and randomization procedures. RCTs provide a highly structured environment, which is considered to possibly have an adherence-fostering effect [21,22]. The efficacy derived from RCTs of internet-based interventions might be overestimated for what can be expected when implementing in routine care, limiting the knowledge base for routine clinical practice [20].

Hence, after establishing the efficacy of an intervention and its subsequent implementation, the so-called *phase IV clinical trials* should follow investigating benefits when implemented as well as potential negative effects implemented [23,24]. Thus, the investigation of the effectiveness of iCBT under routine care conditions is an important part of the evaluation of these services before wide-scale adoption.

Andersson and Hedman [25] reported on the effectiveness of iCBT within four controlled trials and eight open studies for a multitude of mental health problems, indicating that it might be possible to replicate the findings of controlled efficacy trials on guided iCBT in clinical practice. However, in that review, both routine care and RCTs were included, and only eight studies reported effects when the service was delivered under routine conditions. Recently, Andrews et al [15] reported the results of computer-based treatments of depression, panic disorder, generalized anxiety disorder, and social phobia in randomized trials. They also identified eight studies on internet-based treatments in routine clinical practice when delivered outside of a randomized trial reporting an average effect size of g=1.07 across all 4 disorders [15]. However, since then, many more studies have been published. In addition, this review did not specifically try to identify nonrandomized trials, possibly leading to unidentified articles. Additionally, they did not provide disorder-specific results, specific results on guided treatments by mixing guided and unguided treatments, and did not investigate the acceptability and potential negative effects.

The aim of this study was to examine the effects of guided iCBT for the treatment of depression and anxiety under routine care conditions on symptom change, acceptability (uptake, participants’ characteristics, adherence, and satisfaction), and predictors of negative effects (deterioration and side effects).

## Methods

We report this meta-analysis in accordance with the Preferred Reporting Items for Systematic Reviews and Meta-Analyses guidelines (Multimedia Appendix 1) [26]. This meta-analysis was registered at international prospective register of systematic reviews (PROSPERO; trial registration: CRD42018095704).

We searched PubMed, PsychINFO, and the Cochrane library. We used index terms and text words associated with depression and anxiety, internet interventions, and routine care (for a full search string, the reader is referred to Multimedia Appendix 2). Furthermore, we contacted experts in the field to ask whether they were aware of the studies that we did not identify through our systematic literature searches. Furthermore, we conducted reference tracking on the identified studies and previous meta-analyses in the field [5,14,15,27]. The resulting hits of our literature searches were screened on titles and abstracts by 2 independent reviewers (AE and CV). Studies considered as potentially relevant were screened on full text by the same reviewers independently. In case of disagreement, the opinion of a third senior reviewer (DE) was sought.

### Inclusion Criteria

We included studies that (1) examined the effectiveness of a guided or blended iCBT in (2) treating adults with depressive and/or anxiety symptoms (3) under routine care conditions (4) in a pre-post design. We followed the inclusion of adults and older adolescents (aged >16 years) within the treatment provision for adults, as reported in the original studies.

We defined routine care studies as effectiveness studies, which were conducted as nonrandomized clinical trials in settings equal to or representative of routine practice [28]. The definition of routine care differs between countries and health care systems and describes the established way of working at the time of the original study. Depression and anxiety symptoms had to be established based on cutoff scores on self-report outcome measures, clinical diagnosis, or expert opinion. The definition of anxiety symptoms is based on the Diagnostic and Statistical Manual of Mental Disorders IV classification criteria for anxiety disorders. Furthermore, the interventions were considered as guided when the guidance was related to the therapeutic content [29] and as blended when the internet-based intervention was combined with face-to-face elements in one integrated standardized treatment protocol [27]. Guidance can be delivered via email, a secure message system, telephone, or face-to-face contact and via video or face-to-face contact in blended treatments. Finally, both disorder-specific and transdiagnostic interventions (targeted at both depression and anxiety simultaneously) were included.

### Exclusion Criteria

We excluded studies that did not (1) focus primarily on anxiety or depression or (2) provide sufficient data for the calculation of the effect sizes. Studies were also excluded if (1) the service had only been provided as part of a research study, (2) the study could be considered as a feasibility or pilot trial, or (3) patients were randomized at an individual level. However, cluster randomized trials were considered eligible, in which randomization took place not on an individual level but, for example, on a health care institution level. For the definition of feasibility and pilot trials, we followed the NIHR Evaluation, Trials and Studies Coordinating Centre definition of pilot and feasibility trials [30], as recommended by Arain et al [31]. Feasibility trials were defined as “pieces of research done before a main study” (designed around the research question “Can this be done?”), and pilot studies are defined as a version of the main study that is “run in miniature to test whether the components of the main study can all work together” [31]. Additionally, we only included studies published in English, German, or Dutch language.

### Data Extraction

We extracted data related to study and iCBT service–related characteristics, acceptability, effects on symptom change, negative effects, and data related to the risk of bias of reported results.

Study characteristics included the year of publication, the country in which the study was conducted, the year of data collection, sample size, eligibility criteria (establishment of depression and/or anxiety diagnosis at baseline [standardized clinical interview, cutoff on standardized questionnaire, and clinical judgment], inclusion of severe cases [yes/no], and exclusion of cases with suicidal ideation [yes/no], and approach to data analysis [ITT/completer]).

iCBT service–related characteristics included intervention name, the symptoms targeted (depression and/or anxiety), if it was a blended treatment (yes/no), evidence base for the used intervention (positive results based on at least one randomized clinical trial [yes/no]), and whether it was a symptom-specific or transdiagnostic treatment. We also included the recruitment pathway (open community, clinical referral, and both), the number of planned intervention modules, guidance focus (content-focused, motivational-focused, and administrative-focused), guidance delivery format (synchronous vs asynchronous, within the treatment platform vs outside, eg, by email), and guidance moment (as a reaction to an action of the participant [eg, after the participant finished a session, as a reaction to a nonresponse] or planned in different intervals [eg, weekly or biweekly]). Furthermore, we included guides’ professional training (psychotherapist, psychiatrist, general practitioner [GP], psychologist, psychological registrar, nurse, coach [with lived experience]), training of professionals in iCBT (yes/no), supervision of professionals by a trained clinician (yes/no), the planned and actual intensity of guidance in minutes, and if there was a guidance manual provided (yes/no). Additional information on whether a standardized procedure in the case of symptom deterioration and crisis (yes/no) has been established was included.

Acceptability data were extracted with regard to uptake (the number of people screened for the service, people included, and participants starting the treatment), patient characteristics (age and gender), average symptom severity at baseline, adherence (ie, number of completed modules), mean treatment duration in weeks, and participant satisfaction. Negative effects were extracted with regard to average effects on symptom deterioration, other side effects, and reports of specific subgroups at risk for symptom deterioration.

Two reviewers (AE and CV) extracted the data independently, and data sets were merged. Differences and points of uncertainty were discussed and checked by returning to the original article and in some cases to the authors of the respective article.

### Risk of Bias Assessment

Assessing the quality of naturalistic observational studies is challenging as there is no widely accepted tool in doing so [32]. Moreover, established guidelines for the quality assessment of nonrandomized trials are only partially applicable, as they assume comparisons of interventions (Risk Of Bias In Nonrandomized Studies of Interventions-I [33]). Thus, in this study, we selected and adapted criteria from two quality assessment tools [33,34] and adapted them to this study’s purposes to evaluate the risk of bias of the included studies. For the present risk of bias assessment, we discussed the aforementioned assessment tools among all coauthors of this manuscript and derived the analysis criteria described in Multimedia Appendix 3 [35]. As a result, we evaluated (1) researcher allegiance (defined as the first or last author of the study also being the first or last author of the intervention development or efficacy paper), (2) confounding introduced by patients’ participation in other treatments, (3) confounding introduced by significant confounding variables identified within the individual study (meaning any predictors included such as age, guidance, or recruitment pathway), (4) selection bias introduced by the study population (ie, have the studies only reported on completer data), and (5) selective outcome reporting in comparison with the study protocol or diagnostic measures administered as mentioned in the original studies’ methods section. A description of the risk of bias assessment and its operationalization can be found in Multimedia Appendix 3. With regard to *Researcher Allegiance*, we chose the above definition after consideration among the authors and evaluated a study as at high risk of researcher allegiance when the first or last author of the study was also involved in the development of the treatment manual of the psychotherapy involved or the reporting on the interventions’ efficacy. Although the validity of other indicators has been questioned, the involvement of a researcher in developing the treatment under investigation can be considered a valid indicator of potential researcher allegiance [36].

Two reviewers evaluated the quality of the included studies independently (AE and CV). Any disagreement between reviewers was solved by a thorough discussion. If the disagreement could not be resolved, a third senior reviewer was consulted (DE).

### Statistical Analysis

Our primary outcome was the reduction of depressive or anxiety symptoms from pre- to posttest assessment. We calculated the difference in depression and anxiety symptoms between pre-post assessment divided by the weighted, pooled standard deviation (Hedges’ *g*). We have chosen Hedges’ *g* because it allows for small sample size bias correction [37]. As we expected considerable heterogeneity among the studies, we used the random effects model. As a rule of thumb, effect sizes of 0.8 can be viewed as large, 0.5 as moderate, and 0.2 as small [38]. In our main analysis, we included mixed depression and/or anxiety studies into the separate depression and anxiety data sets. Statistical analysis was conducted using the Comprehensive Meta-analysis program (version 2.2.2), and pooled proportions were calculated with R [39] package *meta* [40].

To calculate heterogeneity, we used the I^2^-statistic and its 95% CIs as an indicator of heterogeneity in percentages. Heterogeneity was interpreted as low, moderate, and high when 25%, 50%, and 75%, respectively.

We also included the correlations of the used pre-post measures using the mean of 0.76, where none was provided for depression and 0.59 for anxiety following the study by Balk et al [41]. We also conducted sensitivity analyses for correlations set to 0.00, 0.75, and 0.99 to examine the robustness of our findings [42]. We also calculated the prediction interval, which estimates where the true effects are to be expected for 95% of similar studies that might be conducted in the future [43].

As we expected high heterogeneity, we conducted several subgroup analyses to investigate its possible sources. The examined subgroups were related to the method of analysis, time to post assessment, recruitment pathways, disorders, guidance moment (specific timing or as a reaction), guidance modality (email, message, and synchronous), guide profession (with or without specific CBT training), supervision provided (yes/no), guide training provided (yes/no), intervention manual provided (yes/no), approach to data analysis (ITT/completer), and diagnostic method (interview/questionnaire). Subgroup analyses were only carried out with regard to the effects on symptom change. We used the mixed effects model, testing pooled studies within subgroups with random effects models while testing for significant differences between those subgroups with fixed effects models. We only conducted subgroup analysis if the number of studies per category was not less than three. If necessary, we combined predefined subgroups to achieve the necessary group size.

Finally, we conducted meta-regression analyses for the continuous variables, examining the duration of the treatment as a predictor of treatment outcome as well as guidance time, number of contacts, number of sessions completed, and the percentage of treatment completers.

Regarding uptake, we calculated the proportion of (1) included people based on the number of people screened, (2) starters based on the number of people being screened, and (3) starters based on the number of people included. Adherence was analyzed by calculating the percentage of modules completed based on the average number of sessions that were completed by the participants divided by the planned total number of sessions. We also coded the percentage of intervention completers for a 100% completion rate. Additionally, we pooled the age and gender distribution as well as participant satisfaction extracted from original studies. Furthermore, we pooled the percentage of individuals reported to show symptom deterioration (defined as a negative reliable change in the reported outcome), the deterioration rates, reported in the original study.

Publication bias was examined by inspecting the funnel plot [44] and conducting the Egger test of the intercept with a one-tailed significance level of α=.05 [45]. In addition, we used Duval and Tweedie’s trim and fill procedure [46] to adjust the effect size for missing studies.

## Results

### Study Selection

**Figure 1:**
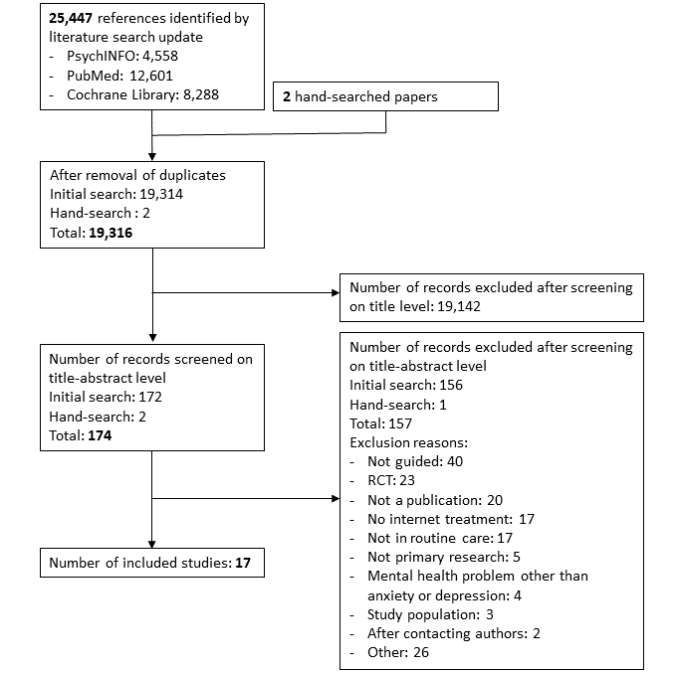
Study inclusion.

A systematic literature search was performed on January 30, 2019. This search resulted in 25,447 citations. After removal of duplicates, 19,316 citations remained for the title and abstract assessment and 174 after the exclusion due to title and abstract. A total of 17 studies fulfilled the eligibility criteria. The full references of the included studies are listed in Multimedia Appendix 4 (Etzelmueller et al, unpublished data, 2020) [47-63]. The study selection process is illustrated in Figure 1.

### Study Characteristics

Table 1 presents the characteristics of the included studies. Seventeen studies (n=12,096 participants) reporting on the outcomes of the treatment for depression and anxiety were included. Seven of the 17 studies reported multiple groups, of which 5 combined results on multiple treatments in the published study and 2 reported distinct forms of guidance within the same treatment and setting without randomizing patients on an individual level. Of the resulting 30 groups, 8 groups focused on depression, 17 on anxiety, and 5 on both depression and anxiety. We included studies reporting on both depression and anxiety in both the depression and anxiety analyses. Of the included studies, 46.7% (*k*=14/30, *k*_Dep_=4, *k*_Anx_=10) administered diagnostic interviews, 36.7% (*k*=11/30; *k*_Dep_=6/13, *k*_Anx_=7/22) self-reports, and 16.7% (*k*=5/30; *k*_Dep_=3/13, *k*_Anx_=2/22) clinical judgment in their diagnostic process. Of the included studies, 30.0% (*k*=9/30; *k*_Dep_=4/13, *k*_Anx_=5/22) administered a cutoff criterion for the self-report questionnaires. Ten studies (33.3%, *k*=10/30; *k*_Dep_=6/13, *k*_Anx_=5/22) included patients who did not meet the criteria for a clinical diagnosis of depression or anxiety. A total of 9.1% of anxiety studies (*k*=2/22) excluded cases with severe symptom severity; all depression studies allowed patients with severe depression severity to be included. Of the included studies, 40.0% (*k*=12/30; *k*_Dep_=4/13, *k*_Anx_=9/22) specifically stated that the patients had to be diagnosed with a clinical depression and/or anxiety disorder to follow the iCBT intervention. The rest of the studies did not specify whether the patients had clinical depression and/or anxiety. Of the included studies, 73.0% (*k*=22/30; *k*_Anx_=9/13, *k*_Dep_=18/22) stated that suicidal ideation or intent was a reason for excluding the patient from the service.

**Table 1:** Study characteristics.

Publication and substudy		Year of publication	Data collection^a^	Country	Sample size	Diagnosis conducted	Diagnostic criterion	Inclusion of severe cases	Exclusion: suicidal ideation^b^
**Aydos et al (2009) [30]**									
	N/A^c^	2009	N/A	Australia	17	Interview (MINI^d^)	Clinical	Yes	No
**Alaoui et al (2015) [56]**									
	N/A	2015	2009-2013	Sweden	653	Interview (MINI)	Clinical	Yes	Yes
**Etzelmueller et al (unpublished data, 2020)**									
	N/A	N/A	2014-2017	Germany	349	Self-report (Patient health Questionnaire; PHQ8>10)	Clinical and subclinical	No	No
**Gellatly et al (2018) [57]**									
	N/A	2018	2013-2015	United Kingdom	724	Clinical judgment	Caseness^e^	No	No
**Hadjistavropoulos et al (2014) [59]**									
	GAD^f^	2014	2010-2013	Canada	107	Interview (MINI) + GAD7>5	Clinical and subclinical	No	Yes
	Depression	2014	2010-2013	Canada	80	Interview (MINI) + PHQ>5	Clinical and subclinical	No	Yes
	Panic disorder	2014	2010-2013	Canada	25	Interview (MINI) + Panic Disorder Severity Scale-Self Report; PDSS-SR>8	Clinical and subclinical	No	Yes
**Hadjistavropoulos et al (2016) [58]**									
	Specialized care	2016	2013-2015	Canada	260	Self-report (Anxiety and depression checklist; K10≥17)	Clinical	No	Yes
	Nonspecialized care	2016	2013-2015	Canada	198	Self-report (K10≥17)	Clinical	No	Yes
**Hedman et al (2013) [60]**									
	N/A	2013	2007-2012	Sweden	1203	Interview (MINI)	Clinical	No	No
**Hedman et al (2014) [48]**									
	N/A	2014	2007-2013	Sweden	570	Interview (MINI)	Clinical	No	No
**Marks et al (2003) [49]**									
	Phobia/panic	2003	N/A	United Kingdom	27	Clinical judgment (International Statistical Classification of Diseases and Related Health Problems; ICD10)	Clinical	No	Yes
	Depression^g^	2003	N/A	United Kingdom	38	Clinical judgment (ICD10)	Clinical	No	Yes
	Anxiety/depression	2003	N/A	United Kingdom	33	Clinical judgment (ICD10)	Clinical	No	Yes
	OCD^h^	2003	N/A	United Kingdom	9	Clinical judgment (ICD10)	Clinical	No	Yes
**Mathiasen et al (2018) [61]**									
	Depression	2018	2016-2017	Denmark	60	Interview	Clinical	No	Yes
	Anxiety	2018	2016-2017	Denmark	143	Interview	Clinical	No	Yes
**Morrison et al (2014) [54]**									
	N/A	2014	2012	United Kingdom	12	Self-report and clinical judgment^i^	Caseness^e^	No	No
**Nordgreen et al (2018) [62,63]**									
	N/A	2018	2014-2016	Norway	124	Interview (MINI)	Clinical	No	Yes
**Nordgreen et al (2018^b^)**									
	N/A	2018	N/A	Norway	169	Interview (MINI)	Clinical	No	Yes
**Ruwaard et al (2012) [50]**									
	Depression	2012	2002-2008	The Netherlands	405	Interview (N/A)	Clinical	No	Yes
	Panic disorder	2012	2002-2008	The Netherlands	136	Interview (N/A)	Clinical	No	Yes
	PTSD^j^	2012	2002-2008	The Netherlands	477	Interview (N/A)	Clinical	No	Yes
**Shandley et al (2008) [51]**									
	general practitioner–guided	2008	N/A	Australia	51	Self-report and interview	Clinical	No	No
	Therapist-guided	2008	N/A	Australia	41	Self-report and interview	Clinical	No	No
**Titov et al (2017) [53]**									
	Depression	2017	2013-2016	Australia	5427	Self-report	Principal complaint	No	Yes
	Depression^k^	2017	2013-2016	Australia	516	Self-report	Principal complaint	No	Yes
	OCD	2017	2013-2016	Australia	69	Self-report	Principal complaint	No	Yes
	PTSD	2017	2013-2016	Australia	137	Self-report	Principal complaint	No	Yes
**Yu et al (2018) [52]**									
	N/A	2018	NA	United States	63	Self-report (GAD7≥5)	Clinical	No	Yes

^a^Data collection period.

^b^Exclusion of cases with suicidal ideation.

^c^N/A: not applicable.

^d^MINI: mini-international neuropsychiatric interview.

^e^Caseness for PHQ-9 refers to a person reporting scores of 10 on the PHQ-9.

^f^GAD: Generalized anxiety disorder.

^g^Transdiagnostic treatment for depression.

^h^OCD: obsessive-compulsive disorder.

^i^Participants were initially identified as suitable to receive a low-intensity intervention for depression or low mood through the triage of a patient’s self-assessment form by team leaders, all of whom were qualified CBT therapists. Patients then had an initial assessment with a psychological well-being practitioner who considered a person’s suitability for MindBalance in reference to the patient’s identified difficulties, goals, and the studies’ inclusion and exclusion criteria (inclusion: to receive treatment of depression with little or no comorbid anxiety, appropriate for guided self-help in a primary-care setting as determined by current [...] procedures).

^j^PTSD: posttraumatic stress disorder.

^k^Depression treatment for older adults.

### iCBT Service–Related Characteristics

Of the studies, 26.3% (*k*=5/19) used transdiagnostic interventions, and all others utilized disorder-specific interventions. We did not identify any blended treatments.

Of the included studies, 31.6% (*k*=6/19) involved clinical referrals in their service pathway, 26.3% (*k*=5/19) did not involve referrals, but only included patients through the general community, whereas 42.1% (*k*=8/19) were recruited in both a community and clinical setting.

On average, iCBT treatments included 8.00 sessions (SD 2.62; *k*=26; depression: *k*=11; mean 8.09, SD 2.84; anxiety: *k*=19; mean 8.00, SD 2.81).

With regard to guidance, 46.7% of the studies (*k*=14/30; *k*_Dep_=5/13, *k*_Anx_=11/22) stated that guidance focused mainly on motivational and 16.7% (*k*=5/30; *k*_De_=4/13, *k*_Anx_=4/22) on administrative aspects. All included studies provided feedback on the content of participants who completed the sessions. Of the studies, 73.3% (*k*=22/30; *k*_Dep_=10/13, *k*_Anx_=14/22) used asynchronous contact methods for communication between participants and guides, 30.0% (*k*=9/30; *k*_Dep_=7/13, *k*_Anx_=4/22) used build-in message systems, and 16.7% (*k*=5/30; *k*_Dep_=1/13, *k*_Anx_=5/22) used emails. Of the studies, 23.3% (*k*=7/30; *k_Dep_*=3/13, *k*_Anx_=7/22) used synchronous contact via telephone contacts, of which one would also use face-to-face contacts. Of the studies, 16.7% (*k*=5/30; *k*_Dep_=1/13, *k_Anx_*=4/22) stated that they provided feedback as a reaction following a participant’s action and 30.0% (*k*=9/30; *k*_Dep_=4/13, *k*_Anx_=6/22) in specific time intervals, weekly or biweekly.

Of the studies, 23.3% (*k*=7/30; *k*_Dep_=4/13, *k*_Anx_=6/22) only involved guides not trained in CBT, whereas the other studies included specifically trained professionals, such as psychotherapists, psychiatrists, GPs, or psychologists. Of the studies, 40.0% (*k*=12/30; *k*_Dep_=7/13, *k*_Anx_=10/22) stated that they provided specific training for the provision of the iCBT intervention to the guides, and 63.3% (*k*=19/30; *k*_Dep_=9/13, *k*_Anx_=13/22) provided supervision to the guiding participants. A total of 26.7% of the studies (*k*=8/30; *k*_Dep_=4/13, *k*_Anx_=4/22) reported having provided an iCBT intervention manual. The average reported guidance time was 148.50 min (SD 146.99; *k*=12; 95% CI 92.87-204.12; depression: *k*=4, mean 82.44 min (SD 81.14), 95% CI 45.29-119.60; anxiety: *k*=9; mean 157.60 min, SD 108.10; 95% CI 92.33-222.86). The pooled results are presented in Table 2.

Nine studies (47.4%; *k*=19) reported safety measures in cases of suicidality, suicidal ideation, or severe symptom deterioration. They mention monitoring systems (*k*=2), risk alerts (*k*=1), or reviewing the participants’ messages (*k*=2) as ways to identify risk. Procedures were triggered in cases of suicidal ideation or suicidality (*k*=9), inactivity or lack of progress (*k*=2), or an increase in symptoms (*k*=2). Ways to mitigate the risk included contacting the participant via telephone (often in contrast to the usual messaging, *k*=4), structured risk assessments (*k*=1), referred to another service (k=6), and the development of a safety plan together with the participant (*k*=1). Information is depicted in Multimedia Appendix 5 [64-101] and Multimedia Appendix 6 [102-120] on the iCBT service–related characteristics.

**Table 2:** Pooled results of iCBT service– and acceptability-related outcomes: guidance time, age, gender, completed sessions, completed components, and deterioration rates.

Groups		Number of studies	Pooled mean (SD)	95% CI	Range
**Guidance time^a^(min)**					
	All studies	12	148.50 (146.99)	92.9-204.1	43.0-378.6
	Depression studies	4	82.44 (290.46)	45.29-119.60	43.0-183.0
	Anxiety studies	9	157.60 (108.10)	92.33-222.86	43.0-378.6
**Age (years)**					
	All studies	29	38.3 (3.02)	37.2-39.4	29.8-43.5
	Depression studies	12	39.0 (2.12)	37.8-40.2	29.0-41.7
	Anxiety studies	21	37.8 (3.04)	36.5-39.2	29.8-43.5
**Gender, female (n, %)**					
	All studies	23	65.4 (20.06)	57.2-72.8	22.2-91.7
	Depression studies	11	70.1 (24.37)	55.7-81.4	22.2-91.7
	Anxiety studies	17	64.3 (17.25)	56.1-71.6	22.2-86.0
**Average percentage of sessions completed**					
	All studies	14	60.6 (6.49)	57.2-72.8	16.7-90.0
	Depression studies	5	62.6 (1.60)	61.2-63.9	16.7-90.0
	Anxiety studies	10	57.3 (1.94)	56.1-58.4	16.7-74.3
**Average percentage of participant completing all treatment components**					
	All studies	26	61.0 (14.83)	55.3-66.9	27.3-82.6
	Depression studies	12	62.8 (13.61)	55.1-70.0	44.0-82.6
	Anxiety studies	18	61.7 (17.75)	53.5-69.3	27.3-82.0
**Deterioration (% deterioration in sample)**					
	All studies	14	2.9 (1.91)	1.9-4.3	1.0-16.6
	Depression studies	5	2.5 (0.34)	2.2-2.9	1.0-12.5
	Anxiety studies	9	3.1 (2.30)	1.6-5.9	1.0-16.6

^a^Excluded study Ruwaard et al [50] as outlier.

### Risk of Bias of the Included Studies

**Figure 2:**
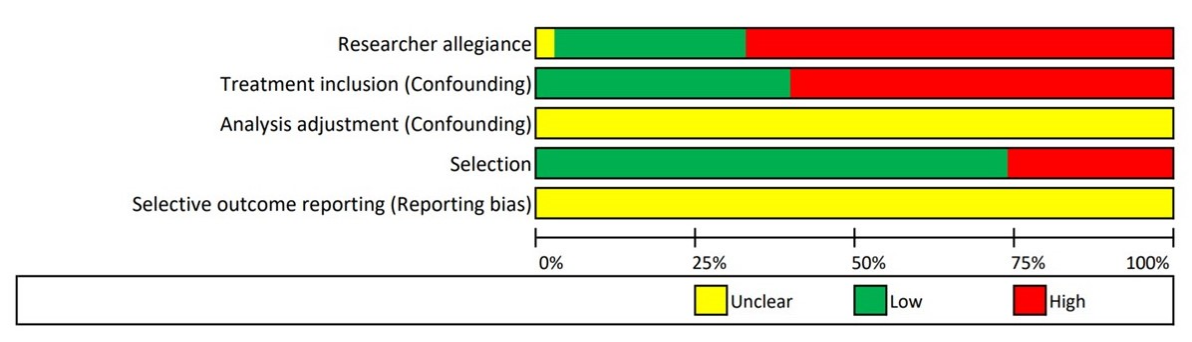
Risk of bias assessment.

The quality of the included studies varied. Of the studies, 67.0% (*k*=20/30) were rated with a high risk of bias on *Researcher Allegiance*. Of the studies, 63.0% (*k*=19/30) did not exclude patients who were participating in other psychotherapeutic treatments (*Treatment Inclusion Confounding*), and none of the studies reported on the adjustment for confounders in the data analysis. Intention-to-treat data could be extracted from 73.3% of the studies (*k*=22/30), and none of the studies were preceded by a published study protocol. The risk of bias assessment is depicted in Figure 2.

### iCBT Service Acceptability

Acceptability data on uptake, participant characteristics across studies, adherence, and participant satisfaction were pooled. All acceptability results are depicted in Multimedia Appendices 6 and 7. The pooled results are presented in Table 2.

#### Uptake

The average proportion of included people based on the number of people screened was 70.2% (*k*=6/30; 95% CI 8.4%-98.4%; range=0.6%-76.0%), the proportion of starters based on the number of people being screened was 48.0% (*k*=10; 95% CI 16.9%-80.8%; range=0.3%- 96.2%), and the proportion of starters based on the number of people included was 73.0% (*k*=7; 95% CI 51.0%-87.6%; *range*=40.6%- 95.9%).

#### Participant Characteristics

The pooled percentage of female participants was 65.4% (*k*=23, 95% CI 57.2%-72.8%; depression: *k*=11, mean 70.1%, 95% CI 55.7%-81.4%; anxiety: *k*=17, mean 64.3%, 95% CI 56.1%-71.6%). The mean age across studies was 38.30 years (*k*=29, 95% CI 37.22-39.37; depression: *k*=12, mean 38.96, 95% CI 37.77-40.15; anxiety: *k*=21, mean 37.83, 95% CI 36.47-39.20).

#### Adherence

The average percentage of sessions completed was 61.2% (*k*=14, 95% CI 54.9%-67.5%; depression: *k*=5, mean 62.6%, 95% CI 61.2%-63.9%; anxiety: *k*=10, mean 57.3%, 95% CI 56.1%-58.4%). The percentage of participant completing all treatment components was 61.3% (*k*=26, 95% CI 55.3%-66.9%; depression: *k*=12, mean 62.8%, 95% CI 55.1%-70.0%; anxiety: *k*=18, mean 61.7%, 95% CI 53.5%-69.3%).

#### Participant Satisfaction

Of the 17 studies, 10 (58.8%) reported participants’ satisfaction. Participant satisfaction outcomes were reported inconsistently, using varying measures and different reporting forms. Therefore, these data could not be pooled, but the detailed results and the data extracted on patient satisfaction are depicted in Multimedia Appendix 7 [81,121-123]. Within the studies reporting participants’ satisfaction, five studies reported a high and four a very high participants’ satisfaction.

### Effects of iCBT on Symptom Change

#### Depression

Effect sizes for changes in depression severity ranged from 0.66 to 1.88 (Hedges’ *g, k=*13 studies), with 1 study (7.7%) reporting a moderate and 12 (92.3%) a large effect size.

The average pre-post effect size of all depression treatments was *g*=1.18 (95% CI 1.06-1.29), which can be considered a large effect. Heterogeneity was significant and high (*I^2^*=95%; 95% CI 94-97; *P*<.001). The prediction interval is 0.74-1.62, and we can expect that in 95% of all populations, the true effect size will fall within this range.

**Figure 3:**
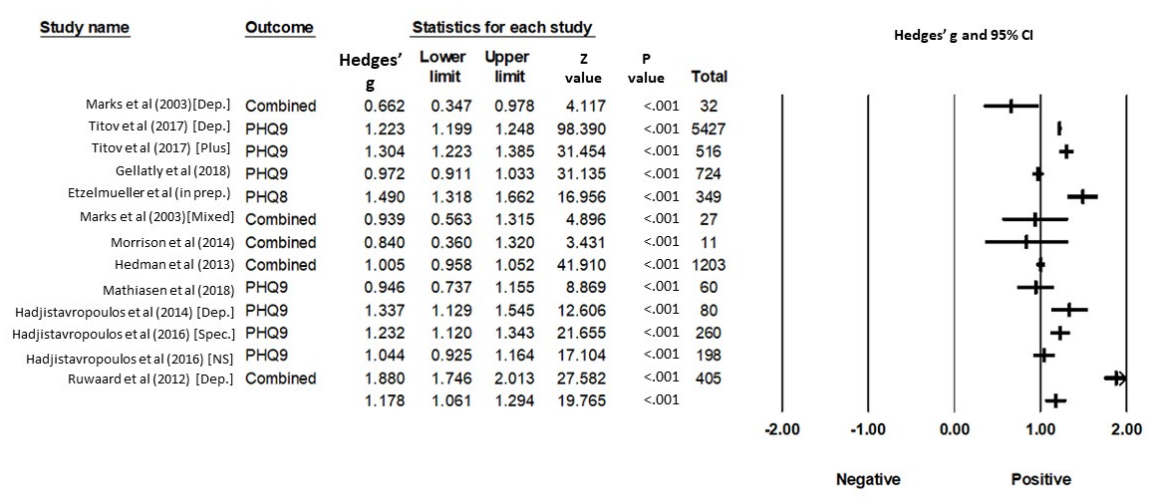
Standardized Effects of iCBT treatments for depression in routine care. Full references are available in Multimedia Appendix 4. Combined: multiple measures for the main outcome have been combined in the analysis; Dep.: depression treatment; Mixed: mixed depression and anxiety treatment; NS: nonspecialized care; PHQ 8: Patient health Questionnaire – 8 Item version; PHQ 9: Patient Health Questionnaire; Plus: depression treatment for older adults; Spec.: specialized care.

The details of these results are shown in Figure 3 and Table 3.

**Table 3:** Meta-analytic comparison of anxiety and depression interventions.

Characteristics		Effect		Heterogeneity	
		g	95% CI	*P* value	I^2^ (95% CI)
**Depression**					
	All studies (n=13)	1.178	1.06-1.29	<.001	95 (94-97)
	Pre-post correlation=0.00	1.236	1.10-1.38	<.001	86 (78-91)
	Pre-post correlation=0.75	1.155	1.04-1.27	<.001	96 (94-97)
	Pre-post correlation=0.99	0.749	0.16-0.88	<.001	100 (100-100)
	Outliers excluded^a^	1.176	1.09-1.26	.001	75 (42-86)
	Without mixed treatments	1.282	1.26-1.44	<.001	89 (84-92)
**Anxiety**					
	All studies (n=20)	0.94	0.83-1.06	<.001	74 (60-83)
	Pre-post correlation=0.00	0.95	0.83-1.07	<.001	93 (91-95)
	Pre-post correlation=0.75	0.93	0.82-1.04	<.001	100 (99-100)
	Pre-post correlation=0.99	0.70	0.62-0.78	<.001	77 (62-86)
	Outliers excluded^b^	0.90	0.81-0.99	<.001	91 (88-95)
	Without mixed treatments	0.95	0.81-1.10	<.001	83 (74-89)
	Without OCD^c^ treatments	0.93	0.81-1.05	<.001	84 (76-90)
	Without PTSD^d^ treatments	0.88	0.78-0.98	<.001	95 (94-97)
	Neither OCD nor PTSD	0.87	0.77-0.98	<.001	86 (78-91)

^a^Three excluded studies [47-49] as well as depression study by Ruwaard et al [50].

^b^Two excluded studies [51,52] as well as posttraumatic stress disorder (PTSD) and panic disorder studies by Ruwaard et al [50] and PTSD study by Titov et al [53].

^c^OCD: obsessive-compulsive disorder.

^d^PTSD: posttraumatic stress disorder.

In this analysis, the pre-post measurement correlation was set to the actual pre-post correlation of the measure (between 0.36 and 0.78). Sensitivity analysis, with correlations set to 0, 0.75, and 0.99, resulted in comparable effect sizes (*g*_Corr=0_=1.24, *I^2^*_Corr=0_=86, 95% CI 78-91; *P*<.001; *g*_Corr=.75_=1.16, *I^2^*_Corr=.75_=96, 95% CI 94-97; *P*<.001), with *g*_Corr=0.99_=0.75 (*I^2^*_Corr=.99_=100, 95% CI 100-100; *P*<.001) resulting in the smallest effect size.

Both the visual inspection of the funnel plot and Egger test (*P*=.90) did not indicate a potential publication bias.

We found five studies to be outliers, defined as not overlapping with the 95% CI of the pooled estimate. Removing these studies [47-49], and the depression group in the study by Ruwaard et al [50], from the analysis did not result in meaningful changes in effect sizes (*g*=1.18, 95% CI 1.09-1.26), but reduced heterogeneity (*I^2^*=75%; 95% CI 42-86; *P*<.001). Removing the mixed anxiety and depression studies did not result in a relevant change in effect size (*g*=1.28; 95% CI 1.13-1.44; *I^2^*=97%; 95% CI 95-98; *P*<.001).

#### Anxiety

For the included anxiety studies (*k*=20), effect sizes ranged from 0.42 to 1.38 (*Hedges’ g*), with 1 study (5.0%) reporting a small, 6 (30.0%) a moderate, and 13 (65.0%) a large effect size.

**Figure 4:**
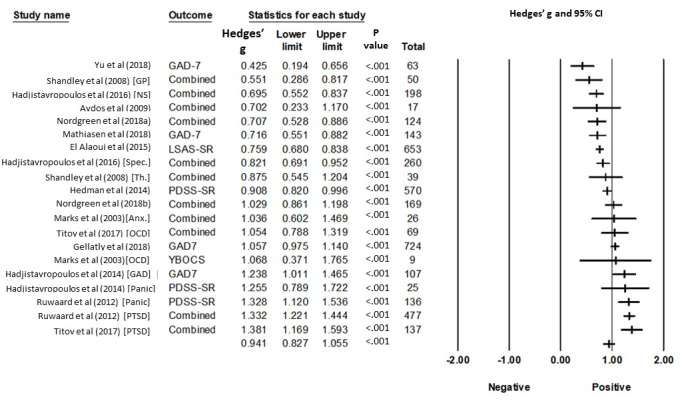
Standardized Effects of iCBT treatments for anxiety in routine care. Marks (2003) is not providing an anxiety measure for the mixed depression and anxiety treatment; therefore, this study has not been included in the analysis. Full references are available in Multimedia Appendix 4. Combined: multiple measures for the main outcome have been combined in the analysis; GAD: generalized anxiety disorder; GP: general practitioner-guided; LSAS: Liebowitz Social Anxiety Scale; NS: nonspecialized care; OCD: obsessive-compulsive disorder; PDSS-SR: Panic Disorder Severity Scale-Self Report; PTSD: posttraumatic stress disorder; Spec.: specialized care; Th.: therapist-guided; YBOCS: Yale-Brown Obsessive Compulsive Scale.

The average pre-post effect size (*Hedges’ g*) of all anxiety interventions, including the interventions that targeted both anxiety and depression, was *g*=0.94 (95% CI 0.83-1.06), which is considered a large effect. Heterogeneity was high (*I^2^*=89, 95% CI 84-92; *P*<.001). The prediction interval is 0.44-1.44, and we can expect that in 95% of all populations, the true effect size will fall within this range. The details of these results are shown in Figure 4 and Table 3.

In the main analysis described above, the pre-post measurement correlation was set to 0.59. Sensitivity analysis with correlations set to 0, 0.75, and 0.99 resulted in comparable effect sizes (*g*_Corr=0_=0.95, *I^2^*_Corr=0_=74, 95% CI 60-83; *P*<.001; *g*_Corr=.75_=0.93, *I^2^*_Corr=.75_=93, 95% CI 91-95; *P*<.001), with *g*_Corr=0.99_=0.70 (*I^2^*_Corr=.99_=99, 95% CI 99-100; *P*<.001) resulting in the smallest effect size.

Both the visual inspection of the funnel plot and Egger test (*P*=.91) did not indicate a potential publication bias.

We found five studies to be outliers, as their results did not overlap with the 95% CI of the pooled estimate. Removing studies [51,52] as well as PTSD and panic disorder studies by Ruwaard et al [50] and PTSD study by Titov et al [53] from the analysis did not influence the result significantly (*g*=0.90; 95% CI 0.81-0.99; *I^2^*=77%, 95% CI 62-86; *p*<.001), but resulted in less, although still high, heterogeneity. Excluding the mixed anxiety and depression studies did not result in a significantly different effect size (*g*=0.99; 95% CI 0.86-1.12; *I^2^*=92%; 95% CI 88-95; *P*<.001). Neither removing OCD treatments (*g*=0.93; 95% CI 0.82-1.05; *I^2^*=90%; 95% CI 86-93; *P*<.001), PTSD treatments (*g*=0.88; 95% CI 0.78-0.98; *I^2^*=83%, 95% CI 74-89; *P*<.001), or both (*g*=0.87; 95% CI 0.77-0.98; *I^2^*=84%; 95% CI 76-90; *P*<.001) resulted in significantly different effect sizes, although lowering the heterogeneity.

### iCBT Negative Effects

Multimedia Appendix 7 [81,121-123] comprises the results of the negative effects. Less than half of the studies reported deterioration rates (k=7; 41%), with an average deterioration rate of 2.9% (k=14, 95% CI 1.9%-4.3%; depression: k=5, mean 2.5%, 95% CI 2.2%-2.9%; anxiety: k=9, mean 3.1%, 95% CI 1.6%-5.9%; the forest plot can be retrieved from the corresponding author). No study reported other negative effects, and one study mentioned that there were no adverse outcomes. No studies have reported the predictors of deterioration or other negative effects.

### Subgroup Analysis for iCBT for the Treatment of Depression, Anxiety, or Mixed Depression and/or Anxiety

Tables 4 and 5 show the results of all examined subgroup analyses. Significant differences between subgroups were found for professional training of coaches, supervision of coaches, and treatment duration for both depression and anxiety studies and for recruitment pathways for depression studies only. Studies evaluating a period of 9 to 13 weeks of treatment duration reported a significant lower effect size (depression: *g*=1.00, 95% CI 0.95-1.05; *I^2^*=0; 95% CI 0-85; anxiety: *g*=0.83, 95% CI 0.72-0.95; *I^2^*=59; 95% CI 9-81) compared with studies with less than 9 (depression: *g*=1.17, 95% CI 1.01-1.32; *I^2^*=95; 95% CI 92-97; anxiety: *g*=1.16, 95% CI 0.97-1.34; *I^2^*=93; 95% CI 61-91) or more than 13 weeks (depression: *g*=1.37, 95% CI 1.00 to −1.74; *I^2^*=97; 95% CI 94-98; anxiety: *g*=0.98, 95% CI 0.78-1.17; *I^2^*=89; 95% CI 78-94). However, the effect sizes within all examined subgroups were high. 

**Table 4:** Subgroup analyses: depression treatments.

Subgroup analysis^a^		Effects			Heterogeneity				Subgroup analysis		
		N^b^	g	95% CI		I^2^	*P* value	I^2^ 95% CI		Q value	*P* value (Q)
**Recruitment pathway^c^**											
	Clinical and community+clinical	8	1.05	0.95-1.14		78	<.001	56-89		7.253	.007
	Community	5	1.38	1.16-1.59		96	<.001	94-98		7.253	.007
**Specific treatment**											
	Mixed treatment	7	1.10	0.98-1.22		93	<.001	87-96		0.736	.39
	Disorder-specific treatment	6	1.27	0.91-1.62		97	<.001	95-98		0.736	.39
**Diagnosis conducted^d^**											
	Interview	7	1.127	0.89-1.35		97	<.001	95-98		0.946	.33
	Questionnaire	5	1.25	1.16-1.34		81	<.001	57-92		0.946	.33
**Clinical cutoff/minimal symptom severity**											
	Yes	4	1.27	1.08-1.45		84	<.001	60-94		0.897	.34
	No	7	1.17	0.98-1.35		96	<.001	94-97		0.897	.34
**Treatment duration**											
	<9 weeks	5	1.17	1.01-1.32		95	<.001	92-97		7.485	.02
	9-13 weeks	4	1.00	0.95-1.05		0	.85	0-85		7.485	.02
	>13 weeks	4	1.37	1.00-1.74		97	<.001	94-98		7.485	.02
**Guide cognitive behavioral therapy training (profession)^e^**											
	Nonprofessional	4	0.92	0.79-1.05		22	.28	0-88		14.151	<.001
	Other	9	1.27	1.14-1.40		96	<.001	94-97		14.151	<.001
**Guide supervision provided**											
	No	4	0.91	0.75-1.08		39	.18	0-79		10.339	<.001
	Yes	9	1.27	1.13-1.41		96	<.001	94-97		10.339	<.001
**Guide training provided**											
	No	6	0.98	0.94-1.02		7	.37	0-76		21.368	<.001
	Yes	7	1.35	1.20-1.51		95	<.001	91-97		21.368	<.001
**Intervention manual provided**											
	No	9	1.039	0.95-1.137		75	<.001	51-87		10.715	<.001
	Yes	4	1.467	1.23-1.71		97	<.001	95-98		10.715	<.001
**Risk of bias—researcher allegiance**											
	High	7	1.252	1.08-1.42		97	<.001	95-98		1.347	.25
	Low	5	1.119	0.99-1.29		70	.01	23-88		1.347	.25
**Risk of bias—confounding (treatment inclusion)**											
	High	7	1.215	1.06-1.43		96	<.001	94-98		0.985	.32
	Low	5	1.105	0.98-1.23		82	<.001	58-92		0.985	.32

^a^Test against “Guidance format: face-to-face vs written guidance,” “Guidance modality: Message, Email, Telephone, F2F,” and “Guide profession” excluded, as there were too few studies included in analysis.

^b^Number of studies.

^c^Only two studies included via the clinical pathway only. We combined the categories “Both, community and clinical” and “clinical” for this analysis.

^d^Excluding one study [54], as this is the only study using clinical judgment without specifying the use of an interview or questionnaire.

^e^We grouped all studies involving guides not specifically trained in delivering cognitive behavioral therapy in the category “non-professional” and studies involving psychiatrists, psychologists, or psychotherapists in their guidance in the category “other.”

**Table 5:** Subgroup analyses: anxiety treatments.

Characteristics		Effect			Heterogeneity			Subgroup analysis		
		N^a^	g	95% CI	I^2^	*P* value	I^2^ 95% CI		Q value	*P* value (Q)
**Recruitment pathway**										
	Clinical	5	0.77	0.53-1.01	91	<.001	81-95		3.340	.19
	Community	7	1.08	0.85-1.31	88	<.001	78-94		3.340	.19
	Community+clinical	8	0.90	0.78-1.01	74	<.001	46-87		3.340	.19
**Specific disorder**										
	Panic	6	0.95	0.71-1.13	91	<.001	64-92		0.053	.82
	Non panic treatments	14	0.92	0.801-1.09	83	<.001	N/A^b^		0.053	.82
**Guidance: modality**										
	Email	7	1.11	0.95-1.26	56	<.001	0-81		4.744	.09
	Message	8	0.88	0.69-1.06	94	<.001	90-96		4.744	.09
	Synchronous (Telephone or face-to-face	5	0.86	0.66-1.10	83	<.001	60-92		4.744	.09
**Guide cognitive behavioral therapy training (profession)^c^**										
	Nonprofessional	4	0.87	0.47-1.27	88	<.001	73-95		0.165	.69
	Other	16	0.96	0.83-1.09	90	<.001	85-93		0.165	.69
**Guidance: moment**										
	Weekly/biweekly	10	0.66	0.73-1.00	74	<.001	0-85		0.174	.67
	Reaction	4	0.83	0.72-0.94	53	<.001	50-86		0.174	.67
**Guide supervision provided**										
	No	6	0.82	0.70-0.94	57	.04	0-83		2.812	.09
	Yes	14	0.9811	0.83-1.13	90	<.001	86-94		2.812	.09
**Guide training provided**										
	No	10	0.80	0.67-0.93	83	<.001	70-90		5.779	.02
	Yes	10	1.07	0.89-1.26	90	<.001	85-94		5.779	.02
**Intervention manual provided**										
	No	16	0.88	0.75-0.94	81	<.001	70-88		37.209	<.001
	Yes	4	1.30	1.19-1.41	29	.24	0-74		37.209	<.001
**Approach to data analysis**										
	Completer	4	1.05	0.98-1.12	0	<.001	0-77		2.796	.096
	ITT	16	0.92	0.78-1.06	91	<.001	86-94		2.796	.096
**Diagnostic method**										
	Interview	15	0.97	0.84-1.06	88	<.001	83-92		0.388	.53
	Questionnaire	5	0.87	0.6-1.14	91	<.001	82-95		0.388	.53
**Treatment duration**										
	<9 weeks	5	1.16	0.97-1.34	83	<.001	61-91		8.686	.01
	9-13 weeks	8	0.83	0.72-0.95	59	.02	9-81		8.686	.01
	>13 weeks	6	0.98	0.78-1.17	89	<.001	78-94		8.686	.01
**Risk of bias—researcher allegiance**										
	High	N/A	0.99	0.83-1.14	90	<.001	0-60		1.613	.20
	Low	N/A	0.82	0.63-1.02	39	<.001	66-92		1.613	.20
**Risk of bias—confounding (treatment inclusion)**										
	High	N/A	1.03	0.89-1.18	89	<.001	83-93		4.852	.03
	Low	N/A	0.82	0.70-0.93	69	<.001	37-84		4.852	.03

^a^Number of studies.

^b^N/A: not applicable.

^c^We grouped all studies involving guides not specifically trained in delivering cognitive behavioral therapy in the category “non-professional,” and studies involving psychiatrists, psychologists, or psychotherapists in their guidance in the category “other.”

Depression studies that recruited in community settings only reported significantly higher effect sizes (*g*=1.37, 95% CI 1.16-1.59; *I^2^*=96; 95% CI 94-98), compared with studies that recruited in clinical or clinical and community settings (*g*=1.05, 95% CI 0.95-1.14; *I^2^*=78; 95% CI 56-89). Across all recruitment pathways, effect sizes were large, but heterogeneity remained high. We did not find this difference in anxiety studies.

Studies only involving guides, not trained in CBT, showed a significantly lower effect size in depression studies (*g*_Non-professional, Depression_=0.92, 95% CI 0.79-1.05; *I ^2^*=22; 95% CI 0-88) than all other studies, including specifically trained professionals (*g*_Other,Depression_=1.27, 95% CI 1.14-1.40; *I^2^*=96; 95% CI 94-97). We did not find this effect in anxiety studies (*g*_Non-professional,Anxiety_=0.87, 95% CI 0.17-1.27; *I^2^*=88; 95% CI 73-95; *g*_Other,Anxiety_=0.96, 95% CI 0.83-1.09; *I^2^*=90; 95% CI 85-93).

Depression studies reporting to having provided supervision to their coaches, trained their professionals, and provided an intervention manual reported a significantly higher effect size (*g*_Supervision_=1.27, 95% CI 1.13-1.41; *I^2^*=96; 95% CI 94-97; *g*_Training_=1.35, 95% CI 1.20-1.51; *I^2^*=95; 95% CI 91-97; g_Manual_=1.47, 95% CI 1.23-1.71; *I^2^*=97; 95% CI 95-98) compared with studies not reporting to provide these (*g*_NoSupervision_=0.91, 95% CI 0.75-1.08; *I^2^*=39; 95% CI 0-79; *g*_NoTraining_=0.98, 95% CI 0.94-1.02; *I^2^*=7; 95% CI 0-76; *g*_NoManual_=1.04, 95% CI 0.95-1.13; *I^2^*=75; 95% CI 51-87). For anxiety studies, we found similar effects for the reporting of training and providing an intervention manual (*g*_Training_=1.07, 95% CI 0.89-1.26; *I^2^*=90; 95% CI 85-94; *g*_Manual_=1.30, 95% CI 1.19-1.41; *I^2^*=29; 95% CI 0-74 compared with *g*_NoTraining_=0.80, 95% CI 0.67-0.93; *I^2^*=83; 95% CI 70-88; *g*_NoManual_=0.88, 95% CI 0.75-0.94; *I^2^*=81; 95% CI 70-88), but not for supervision.

There were no differences between subgroups regarding all other examined subgroups, both for depression and anxiety studies.

Subgroup analyses comparing studies rated with high versus low risk indicated that R*esearcher Allegiance* did not have a significant influence on the estimated effect sizes for neither anxiety nor depression studies. The heterogeneity within the studies reporting a low risk of bias on *Researcher Allegiance* did reveal an *I^2^* of 39 compared with an *I^2^* of 90 for studies reporting a high risk of bias. Moreover, anxiety studies rated as at high risk of *Treatment Inclusion Confounding* had higher estimated effect sizes. This was not replicated in subgroup analyses of interventions targeting depression. Anxiety studies at high risk of *Selection Bias* reported significantly lower effect sizes. Similar outcomes were not replicated in the depression trials.

### Meta-Regression Analysis for iCBT for the Treatment of Anxiety, Depression, or Mixed Depression and Anxiety

Meta-regression analyses indicated that longer treatment duration in depression studies was positively associated with a higher effect (*P*=.02; β=0.03, *R^2^*=0.00). This effect was not found in anxiety studies (*P*=.94). None of the examined variables, that is, guidance time, number of contacts, number of sessions completed, or the percentage of treatment completers, were significantly associated with the observed effect sizes, neither in depression nor anxiety studies.

## Discussion

This study aims to examine the acceptability, effects on symptom change, and negative effects of guided iCBT interventions in treating depression and anxiety in routine care. Regarding the uptake of the service, on average, 70.2% of people screened were not offered inclusion, and of those included, 73.0% started the intervention. The vast majority of participants reached were female, with an average age of 38.3 years, and 61.3% of participants completed the interventions as planned. Reported participant satisfaction was high, although inconsistently reported results did not allow us to pool effects. The average professional guidance time per participant was 133.49 min over the treatment duration. With regard to the effects on symptom change, the results indicated large average reductions for both depression (*g*=1.18; 95% CI 1.06-1.29) and anxiety (*g*=0.94; 95% CI 0.83-1.062). However, the heterogeneity between studies was high. Nevertheless, all examined effect sizes were at least moderate, indicating the intervention’s potential when delivered under routine care conditions with effects ranging from moderate to large. The average deterioration rates were 3.2% for depression and 3.1% for anxiety. Subgroup analyses indicated a range of iCBT service–related characteristics to be associated with the observed treatment effects.

Regarding uptake, we found that many participants who were in contact with the iCBT service did not start the intervention. Pretreatment dropout is hard to assess, and, accordingly, reasons for not starting an iCBT intervention after inclusion have not been discussed in the original publications.

The average age of participants found in this study (mean 38.30) appears to be slightly lower than that reported in RCTs on guided iCBT interventions for the treatment of depression (mean 42.5 [124]) but comparable with reports on the mean age of participants within guided iCBT interventions for the treatment of anxiety [125]. The percentage of females in the routine care study population was higher for depression studies compared with guided iCBT for the treatment of depression [124] and similar to reports on participants in guided iCBT interventions for the treatment of anxiety [125] in experimental settings. As similar distributions between female and male users are reported in face-to-face mental health service utilization [126], this effect might be explained by gender differences in help-seeking behavior than being related to iCBT service–related factors [127] as well as by gender differences in the prevalence of depression and anxiety disorder [128,129]. Future studies should focus on ways to attract men to use iCBT interventions.

The pooled reported percentage of sessions completed, that is, 62.6% in depression and 57.3% in anxiety studies, was lower than that described in meta-analyses on adherence in RCTs on iCBT interventions. Comparing the adherence to iCBT and face-to-face CBT, van Ballegooijen et al [130] reported that on average, participants completed 80.8% of treatment sessions in the iCBT and 83.9% in the face-to-face intervention [130]. Similarly, the percentage of participants completing the treatment as planned was lower (62.8% for depression and 61.7% for anxiety studies) than reported elsewhere [130,131]. These differences might be due to the assumed adherence-fostering effect of randomized controlled settings versus routine care [132]. However, completion rates were reported inconsistently across studies, applying different criteria such as study or treatment completers, including several definitions of treatment completions. To facilitate comparability, literature on iCBT completion should settle on one reporting standard. Further investigation of factors promoting the acceptance of iCBT interventions, also when reporting on effectiveness results in routine care, may lead to a deeper understanding that might foster intervention development and upscaling.

Results on the effectiveness of iCBT (*g*_Depression_=1.18, 95% CI 1.06-1.29 and *g*_Anxiety_=0.94, 95% CI 0.83-1.062) confirm findings of recently published systematic reviews and meta-analyses on RCTs of iCBT for depression and anxiety. Königbauer et al [12] found medium to large pre-post within-group effects ranging between −0.64 and −2.24 for interventions treating clinical depression [12]. To our knowledge, no recent meta-analysis has reported on pre-post effect sizes of studies targeting guided iCBT interventions for the treatment of anxiety. On an individual study level, pre-post effects in randomized trials ranged from 0.54 to 2.40 (please see Multimedia Appendix 8 for references) [133-162] compared with 0.66 to 1.88 in depression and 0.42 to 1.38 (*Hedges’ g*) in anxiety within this analysis.

With regard to randomized pragmatic trials conducted under routine care conditions, Andrews et al [15] examined a sample of 64 papers reporting results of RCTs on the effectiveness of iCBT for the treatment of depression, panic disorder, generalized anxiety disorder, and social phobia in comparison with control groups in routine practice. This review study reported effect sizes for depression, panic disorder, generalized anxiety disorder, and social phobia ranging from *g*=0.67 to 1.31 [15]. The same study identified eight papers investigating the effectiveness of iCBT, reporting an average effect size of *g*=1.07 across the treatment of depression, panic disorder, generalized anxiety disorder, and social phobia [15]. The between-group effects were moderate to large (*g*=0.72; 95% CI 0.60-0.83; *P*<.001; of *I²*=53, 95% CI 31-66) in the most recent meta-analysis of iCBT treatments for anxiety compared with control conditions in reducing symptoms of anxiety in an adult population [13]. Additionally, the results of this study are in line with meta-analytic findings on face-to-face CBT treatments implemented in routine care with pre-post effect size found in randomized trials ranging from *d*=0.69 to 2.28 for depression [28] and *g*=0.73 to 2.59 for anxiety treatments [163].

The results of deterioration rates (3.2% in depression and 3.1% in anxiety studies) were slightly lower, but within the 95% CI of findings based on RCTs for internet-based guided self-help interventions (3.36%) for depression [164] and anxiety (5.8% [165]), and also comparable with deterioration rates in face-to-face psychotherapy for depression [166]. Criteria defining deterioration varied between studies, and unfortunately, neither were reports on other negative effects included in most primary studies nor reported any study predictors of deterioration. This seems of utmost importance to identify those individuals that should potentially be referred to other mental health services. Their investigation is of specific importance within naturalistic study designs and under routine care conditions [164,165,167].

Most evaluated iCBT services for depression (69.2%) excluded severe cases and individuals with suicidal ideation (*k*=9/13) at baseline. However, a large-scale study showed that iCBT services can also result in positive effects on suicidality, reducing the prevalence of suicidal ideation from 50% at baseline to 27% after treatment [168]. In addition, a recent individual patient data meta-analysis on RCTs indicated that guided iCBT also resulted in clinically meaningful results in individuals with severe depression symptomatology [124]. Given that many individuals applying to iCBT services either do not have access to other immediate care or are not willing to utilize alternative treatment services, future studies should explore the balance between potential risk and benefits of opening up those services to populations showing elevated suicidal ideation. In such cases, it seems of utmost importance to monitor potential upcoming crises using standard operating procedures involving trained clinicians and to evaluate treatment success at the end of the service. In case of nonresponse, individuals should be motivated and guided to utilize other mental health care services, if available. Such standardized crisis procedures were only reported to be employed by less than half of the studies included in this review. iCBT services in routine care might profit from clear pathways of referral to other services in cases of nonresponse and symptom deterioration. Furthermore, future research should facilitate our understanding of the effects of routine outcome monitoring in routinely applied iCBT [169], as this monitoring could help evaluate participants’ progress throughout the course of treatment, using standardized outcome measures to elicit clients as part of a measurement-based care delivery approach in routine mental and behavioral health care [170,171].

The finding that treatment outcomes of depression interventions were greater when recruitment was carried out using an open recruitment strategy in a community setting compared with when recruited in a clinical setting is in line with the findings of Romijn et al [13] with regard to randomized pragmatic studies on anxiety disorder treatments. However, in our study, this interaction was only found for depression and could not be confirmed for anxiety disorders. One potential explanation for the difference in effects might be differences in the characteristics of the included patients. There is evidence that iCBT recruiting via open recruitment strategies, such as through web-based channels, might only reach a specific population that is different from those seeking help in a clinical setting [19]. It is often argued that internet interventions might reach individuals that would otherwise not seek treatment or only at a later time point. Given that, for example, the chronicity of depression is associated with worse treatment outcomes [172], the difference in effect might be explained by reaching a population with lower chronicity. However, such an assumption needs to be confirmed in future studies.

Further subgroup analyses indicated that iCBT services for the treatment of depression utilize trained professionals (psychotherapists and psychiatrists) to result in larger pre-post changes compared with iCBT services that used only nonprofessionals not trained in CBT (psychologists without specialized CBT training, nurses, GPs, counselors, coaches, and lived experience coordinators). However, we did not find this effect in the anxiety studies. Moreover, effects in the subgroup of depression studies involving nonprofessionals were large, indicating the potential to deliver iCBT services, for example, in contexts when there might be a shortage of trained clinicians. In cases where nonprofessionals deliver guidance in iCBT services, supervision by trained clinicians, including the availability of professionals for crisis intervention, seems warranted. Further subgroup analyses also indicated that providing supervision to coaches is also associated with higher average treatment effects for depression, but not for anxiety studies. Furthermore, training the professional and providing an intervention manual is positively related to the interventions’ effectiveness. This result must be interpreted with caution as we coded all studies not mentioning supervision, training, or manual provision in their publication as not providing these components. Furthermore, these components do not inform us about actual treatment fidelity. Further research should focus on the effects on treatment outcomes of providing supervision, training, and intervention manuals to professionals working with iCBT interventions in routine care as well as the assessment of treatment fidelity.

Moreover, we did not find a difference in effects on mean symptom change between iCBT services who applied diagnostic interviews for patient allocation versus those that used self-reports only. This is in line with meta-analytic findings from RCTs on guided digital interventions for depression [124] and with studies directly comparing the effectiveness of iCBT services when treatment allocation was based on an automatic web-based assessment versus clinician assessment [173]. This indicates that such services can be used in contexts when implementing services with initial clinician assessment is not possible, without affecting average treatment success. However, it must be noted that although results might not indicate differences on the group level, it might be the case that using web-based assessments only, without a clinical assessment, will overlook relevant diagnostic information that requires immediate attention, such as suicidal risk or an underlying treatment need for comorbid disorders such as PTSD on an individual level.

The strengths of this meta-analysis include the exclusive focus on evaluating iCBT interventions for their acceptability and clinical outcomes under real-world conditions. Unlike previous systematic reviews that mixed efficacy with effectiveness trials, in this review, we focused only on studies conducted in regular care settings. This is important as we strive to report routine care results free from biases possibly being introduced within efficacy studies such as stricter application of protocolized procedures, eligibility criteria, and randomization [19-22]. Moreover, we presented an overview of implementation indicators existing in the included studies that can be used to gain a better understanding of how iCBT can be adopted by regular care services. Nevertheless, the findings of this study should be interpreted with caution due to several limitations.

First, the heterogeneity in our sample was high and significant, illustrating a great variation in the results of the included studies. Thus, we cannot draw firm conclusions regarding the average effect of iCBT in routine care. Moreover, within-group effect sizes do not depict an optimal estimator for the treatment effect because they are not independent of each other and do not account for recovery occurring independent of the treatment, thereby leading to an overestimation of the treatment effect [42]. However, in comparison with and on the basis of the reported efficacy of iCBT interventions established in RCTs, they depict the best available indicator of the effects of iCBT solutions in a routine care environment. Furthermore, we found that treatment duration had a significant influence on treatment effects. This result also supports the hypothesis that findings on pre-post changes in symptom severity might have been influenced by spontaneous or unexplained recovery, which is a common factor in depression [174]. However, our main results are in line with within-group effect sizes found in RCTs, where spontaneous recovery also occurs, and we, therefore, conclude that our effects can be considered substantial. Although heterogeneity was not explained by any other of the examined subgroups, several assumptions can be made regarding its sources. One other explanation for the high heterogeneity might be the influence of contextual factors of observational studies, such as sampling methods, participant characteristics, within-group effect sizes, and differences between the studies in reporting outcomes. It can be hypothesized that a greater harmonization regarding the conduct and reporting of effectiveness studies in routine care could lead to greater comparability of the studies’ results. Another reason for the observed heterogeneity might be the different contexts of regular care facilities across different countries. There is great variability in the degree of e-mental health penetration in different countries. For instance, Australia is considered one of the frontrunners in the e-mental health field, whereas Norway adopted these interventions very recently [175]. Thus, professionals might differ in the way they interact with e-mental health around the world. Finally, the interventions might differ in the way that they have been developed. These results also imply the importance of establishing a firm evidence base for individual iCBT interventions before their larger upscale.

Second, firm conclusions on treatment effects might be biased by studies that also included participants who could also participate in other psychotherapeutic treatments. Meanwhile, the data do not allow conclusions on the percentage of participants receiving additional treatment and represents the routine practice. Additionally, no study has reported adjusting for confounders such as baseline symptom severity, treatment fidelity (provision and use), or changes in the treatment over the course of the studies, which should be considered in future reports on the effects of iCBT in routine care.

Future studies should add to the body of literature on iCBT interventions examined under routine care conditions. Additionally, these studies should not solely focus on the effectiveness of the interventions, but if possible, it would be helpful if they also reported on specific service-, implementation-, and context-related outcomes. One way of achieving this might be through taxonomy and guidelines for the reporting of iCBT effectiveness, implementation, and context outcomes in routine care. In contrast to standards of reporting RCTs, no such international standards exist when it comes to reporting nonrandomized intervention studies. Proctor et al [176] suggested a list of outcomes for implementation-related research, and Hermes et al [177] recently made suggestions on how to build upon these ideas to establish a measurement system for the implementation of behavioral intervention technologies. Moreover, such research should always be discussed and evaluated in the light of the quality criteria established to help all involved stakeholders, patients, practitioners, and decision makers at the local and policy level to identify not only effective but also safe interventions [178].

In conclusion, this study provides further evidence supporting the acceptability and effectiveness of guided iCBT for the treatment of depression and anxiety when implemented in routine care, whereas results on negative effects are less clear. Guided iCBT may be an effective way of overcoming barriers to treatment provision. It may substantially increase the coverage of usual care services and offer an innovative treatment format for the treatment of depression and anxiety.

## Appendix

Multimedia Appendix 1 Prisma Checklist.

Multimedia Appendix 2 Search strategy.

Multimedia Appendix 3 Risk of Bias Assessment Definition.

Multimedia Appendix 4 References of included studies.

Multimedia Appendix 5 E and F - iCBT service-related characteristics.

Multimedia Appendix 6 Uptake and participant characteristics.

Multimedia Appendix 7 Acceptability - Participant satisfaction and negative effects.

Multimedia Appendix 8 References for studies targeting guided iCBT interventions for the treatment of anxiety reporting on pre-post effect sizes.

## Abbreviations

GP: general practitioner
iCBT: internet-based cognitive behavioral therapy
RCT: randomized controlled trial
