# Correction: Effects of Internet-Based Cognitive Behavioral Therapy in Routine Care for Adults in Treatment for Depression and Anxiety: Systematic Review and Meta-Analysis

**DOI:** 10.2196/69127

**Published:** 2025-12-29

**Authors:** Anne Etzelmueller, Christiaan Vis, Eirini Karyotaki, Harald Baumeister, Nickolai Titov, Matthias Berking, Pim Cuijpers, Heleen Riper, David Daniel Ebert

**Affiliations:** 1 Department of Clinical Psychology and Psychotherapy Friedrich-Alexander University Erlangen-Nuremberg Erlangen Germany; 2 GET.ON Institute GmbH Hamburg Germany; 3 Department of Clinical, Neuro-, & Developmental Psychology Faculty of Behavioural and Movement Sciences VU Amsterdam Amsterdam The Netherlands; 4 Mental Health Amsterdam Public Health Research Institute Amsterdam The Netherlands; 5 Department of Global Health and Social Medicine Harvard Medical School, USA Boston, MA United States; 6 Department of Clinical Psychology and Psychotherapy Institute of Psychology and Education, Ulm University Ulm Germany; 7 eCentre Clinic Department of Psychology Macquarie University Sydney Australia; 8 Community Mental Health Centre GGZ inGeest Amsterdam The Netherlands

In “Effects of Internet-Based Cognitive Behavioral Therapy in Routine Care for Adults in Treatment for Depression and Anxiety: Systematic Review and Meta-Analysis” [1] the authors noted two errors.

In the Results section of the abstract, the pooled effect size for depression has been changed from the following:

1.78

The effect size now reads:

1.18

In addition, the eighth column header in Table 1 has been changed from the following:

Inclusion of severe cases

The column header now reads:

Exclusion of severe cases

The corrections will appear in the online version of the paper on the JMIR Publications website, together with the publication of this correction notice. Because these were made after submission to PubMed, PubMed Central, and other full-text repositories, the corrected article has also been resubmitted to those repositories.
