# Supplementary material for: Effects of Internet-Based Cognitive Behavioral Therapy in Routine Care for Adults in Treatment for Depression and Anxiety: Systematic Review and Meta-Analysis
Source: J Med Internet Res. 2020 Aug 31;22(8):e18100. doi: 10.2196/18100 (PMC7490682; doi:10.2196/18100)
Supplement: Multimedia Appendix 2 [file jmir_v22i8e18100_app2.docx]

**Appendix B** Search strategy

Major Depression[MeSH Terms]) OR Depression[MeSH Terms]) OR affective disorder[MeSH Terms]) OR depressi*[Title/Abstract]) OR mood disorder*[Title/Abstract]) OR dysthymi*[Title/Abstract]) OR anxiety[MeSH Terms]) OR anxiety disorder[MeSH Terms]) OR anxiety disorders[MeSH Terms]) OR panic disorder[MeSH Terms]) OR panic attack[MeSH Terms]) OR agoraphobia[MeSH Terms]) OR acute stress disorder[MeSH Terms]) OR obsessive compulsive disorder[MeSH Terms]) OR phobia[MeSH Terms]) OR phobias[MeSH Terms]) OR Trauma[MeSH Terms]) OR ((Trauma and Stressor Related Disorders[MeSH Terms]))) OR PTSD[MeSH Terms]) OR fear[MeSH Terms]) OR Hypochondriasis[MeSH Terms]) OR adjustment disorder[MeSH Terms]) OR anxiet*[Title/Abstract]) OR pani*[Title/Abstract]) OR agoraphobi*[Title/Abstract]) OR phobi*[Title/Abstract]) OR affective disorder*[Title/Abstract]) OR hypochondri*[Title/Abstract])) OR mood disorder[MeSH Terms])) AND (( ((telemedicine[MeSH Terms]) OR Telehealth[MeSH Terms]) OR "therapy, computer-assisted"[MeSH Terms]) OR Computer-Assisted Instruction[MeSH Terms]) OR computer-assisted diagnosis[MeSH Terms]) OR internet[MeSH Terms]) OR mobile phone[MeSH Terms]) OR mobile applications[MeSH Terms]) OR internet*[Title/Abstract]) OR online*[Title/Abstract]) OR web*[Title/Abstract]) OR telemedicine*[Title/Abstract]) OR telehealth*[Title/Abstract]) OR tele-based[Title/Abstract]) OR mobile*[Title/Abstract]) OR computer*[Title/Abstract]) OR ehealth*[Title/Abstract])) AND ((((routine*[Title/Abstract]) OR Health Plan Implementation[MeSH Terms]) OR implement*[Title/Abstract]) OR up-scale) OR uptake[Title/Abstract]) OR Information Dissemination[MeSH Terms]) OR dissemina*[Title/Abstract]) OR normal*[Title/Abstract]) OR Program Evaluation[MeSH Terms]) OR effecti*[Title/Abstract])
