# Supplementary material for: Effects of Internet-Based Cognitive Behavioral Therapy in Routine Care for Adults in Treatment for Depression and Anxiety: Systematic Review and Meta-Analysis
Source: J Med Internet Res. 2020 Aug 31;22(8):e18100. doi: 10.2196/18100 (PMC7490682; doi:10.2196/18100)
Supplement: Multimedia Appendix 3 [file jmir_v22i8e18100_app3.docx]

**Appendix C** Risk of Bias Assessment Definition

| Bias | Definition in current study | Characteristics | Response definition | Response options for risk of bias |
| --- | --- | --- | --- | --- |
| Researcher Allegiance | Researcher allegiance can be defined as a researcher’s “belief in the superiority of a treatment [and] … the superior validity of the theory of change that is associated with the treatment” (Leykin & DeRubis, 2009, p.55). | Researcher allegiance is operationalized by the fact that the study is conducted by the treatment developer and defined as defined as the first or last author of the study also being the first or last author of the intervention development or efficacy paper. In psychotherapy literature, the first authors are the leading authors of the researcher while last authors are usually the principal investigators. So, those mostly involved in research are placed either first or last. | Present = yes = high risk of bias | Low High unclear |
| Confounding | Confounding of intervention effects occurs when one or more prognostic variables (variables that predict the outcome of interest) also predict whether an individual receives one or the other of the interventions of interest. Baseline confounding, which occurs when one or more prognostic variables predicts the intervention received at start of follow up, is likely to be an issue in most “Non-randomised studies of interventions” (NRSI). [This might occur, when Patient’s receive other psychotherapeutic treatment in addition to the investigated iCBT service.]  Time-varying confounding occurs when the intervention received can change over time (for example, if individuals switch between the interventions being compared), and the intervention is not delivered as intended. | Individuals do not switch between interventions, but the level of control of confounders might be low and so additional treatment might vary over time (e.g. patients starts additional CBT treatment, other TAU activities). If the publication indicated that patient’s already receiving some other form of CBT treatment are excluded from the study, we regard this as a low risk of bias. If the publication does not indicate that patient’s already receiving some other form of CBT treatment are excluded from the study, but this is statistically accounted for by the analysis conducted, the risk of bias will be regarded low as well. | The patient is excluded if he is currently in another treatment = yes = low risk of bias  The analysis is adjusted for any additional therapy received by the patient = yes = low risk of bias | Low High unclear |
|  |  | Have the studies adjusted for confounders in the data analysis | Adjustment = yes = low risk of bias | Low High unclear |
| Selection | When exclusion of some eligible participants, or the post measure, is associated with the interventions provided and outcome even if the true effects of the interventions are identical. | The analysis population will have an influence on effect-sizes and the risk of bias. If an analysis was conducted with all patients intended to be treated, we regard a low risk of bias. | ITT = yes = low risk of bias | Low High unclear |
| Selective outcome reporting | Selective reporting will lead to bias if it is based on the direction, magnitude or statistical significance of intervention effect estimates. Selective outcome reporting occurs when an effect estimate for a particular outcome measurement is selected from among multiple measurements, for example a measurement made at one of a number of time points or based on one of multiple depression/anxiety scales. | Selective outcome reporting will be investigated by the comparison of a published study protocol and the results reported with regards to a) main outcome, b) measures, and c) analysis method. | Selective reporting in comparison to study protocol = yes = high risk of bias  Selective reporting in comparison to study protocol = no = low risk of bias  No study protocol available = unclear risk of bias | Low High unclear |
