# Supplementary material for: Effects of Internet-Based Cognitive Behavioral Therapy in Routine Care for Adults in Treatment for Depression and Anxiety: Systematic Review and Meta-Analysis
Source: J Med Internet Res. 2020 Aug 31;22(8):e18100. doi: 10.2196/18100 (PMC7490682; doi:10.2196/18100)
Supplement: Multimedia Appendix 4 [file jmir_v22i8e18100_app4.docx]

**Appendix D** References of included studies

1. Aydos L, Titov N, Andrews G. Shyness 5: The clinical effectiveness of Internet-based clinician-assisted treatment of social phobia. Australas Psychiatry. 2009;17(6):488-492. doi:10.1080/10398560903284943
2. El Alaoui S, Hedman E, Kaldo V, et al. Effectiveness of Internet-Based Cognitive – Behavior Therapy for Social Anxiety Disorder in Clinical Psychiatry. 2015;83(5):902-914.
3. Etzelmueller A, Heber E, Horvath H, Radkovsky A, Zimmermann J, Ebert DD. The evaluation of the GET.ON nationwide online-only treatment service for depression and stress related symptom in Germany. In prep.
4. Gellatly J, Chisnall L, Seccombe N, Ragan K, Lidbetter N, Cavanagh K. @Home eTherapy Service for People with Common Mental Health Problems: An Evaluation. Behav Cogn Psychother. 2018;46(1):115-120. doi:10.1017/S1352465817000297
5. Hadjistavropoulos HD, Nugent MM, Alberts NM, Staples L, Dear BF, Titov N. Transdiagnostic Internet-delivered cognitive behaviour therapy in Canada: An open trial comparing results of a specialized online clinic and nonspecialized community clinics. J Anxiety Disord. 2016;42:19-29. doi:10.1016/J.JANXDIS.2016.05.006
6. Hadjistavropoulos HD, Pugh NE, Nugent MM, et al. Therapist-assisted Internet-delivered cognitive behavior therapy for depression and anxiety: Translating evidence into clinical practice. J Anxiety Disord. 2014;28(8):884-893. doi:10.1016/J.JANXDIS.2014.09.018
7. Hedman E, Ljótsson B, Kaldo V, et al. Effectiveness of Internet-based cognitive behaviour therapy for depression in routine psychiatric care. J Affect Disord. 2014;155(1):49-58. doi:10.1016/j.jad.2013.10.023
8. Hedman E, Ljótsson B, Rück C, et al. Effectiveness of Internet-based cognitive behaviour therapy for panic disorder in routine psychiatric care. Acta Psychiatr Scand. 2013;128(6):457-467. doi:10.1111/acps.12079
9. Marks IM, Mataix-Cols D, Kenwright M, Cameron R, Hirsch S, Gega L. Pragmatic evaluation of computer-aided self-help for anxiety and depression. Br J Psychiatry. 2003;183(01):57-65. doi:10.1192/bjp.183.1.57
10. Mathiasen K, Riper H, Andersen TE, Roessler KK. Guided Internet-Based Cognitive Behavioral Therapy for Adult Depression and Anxiety in Routine Secondary Care: Observational Study. J Med Internet Res. 2018;20(11):e10927. doi:10.2196/10927
11. Morrison C, Walker G, Ruggeri K, Hacker Hughes J. An implementation pilot of the MindBalance web-based intervention for depression in three IAPT services. Cogn Behav Ther. 2014;7:e15. doi:10.1017/S1754470X14000221
12. Nordgreen T, Gjestad R, Andersson G, Carlbring P, Havik OE. The effectiveness of guided internet-based cognitive behavioral therapy for social anxiety disorder in a routine care setting. Internet Interv. 2018;13:24-29. doi:10.1016/J.INVENT.2018.05.003
13. Nordgreen T, Gjestad R, Andersson G, Carlbring P, Havik OE. The implementation of guided Internet-based cognitive behaviour therapy for panic disorder in a routine-care setting: effectiveness and implementation efforts. Cogn Behav Ther. 2018;47(1):62-75. doi:10.1080/16506073.2017.1348389
14. Ruwaard J, Lange A, Schrieken B, Dolan C V., Emmelkamp P. The Effectiveness of Online Cognitive Behavioral Treatment in Routine Clinical Practice. Botbol M, ed. PLoS One. 2012;7(7):e40089. doi:10.1371/journal.pone.0040089
15. Shandley K, Austin DW, Klein B, et al. Therapist-assisted, internet-based treatment for panic disorder: Can general practitioners achieve comparable patient outcomes to psychologists? J Med Internet Res. 2008;10(2):e14. doi:10.2196/jmir.1033
16. Titov N, Dear BF, Staples LG, et al. The first 30 months of the MindSpot Clinic: Evaluation of a national e-mental health service against project objectives. Aust N Z J Psychiatry. 2017;51(12):1227-1239. doi:10.1177/0004867416671598
17. Yu JS, Szigethy E, Wallace M, Solano F, Oser M. Implementation of a Guided, Digital Cognitive Behavioral Program for Anxiety in Primary Care: Preliminary Findings of Engagement and Effectiveness. Telemed e-Health. 2018;24(11):970-978. doi:10.1089/tmj.2017.0280
