# Supplementary material for: Effects of Internet-Based Cognitive Behavioral Therapy in Routine Care for Adults in Treatment for Depression and Anxiety: Systematic Review and Meta-Analysis
Source: J Med Internet Res. 2020 Aug 31;22(8):e18100. doi: 10.2196/18100 (PMC7490682; doi:10.2196/18100)
Supplement: Multimedia Appendix 5 [file jmir_v22i8e18100_app5.docx]

**Appendix E** iCBT service-related characteristics – Part I

| Publication | Sub-Study | Intervention name | Disorder | Blended treatment | Evidence Base [A] | Transdiagnostic or symptom specific treatment | Recruitment pathway | Number of planned intervention modules [B] |
| --- | --- | --- | --- | --- | --- | --- | --- | --- |
| Aydos et al., (2009) |  | Shyness Programm | Social Phobia | No | Yes ^1-3^ | Specific | Community | 6 |
| Alaoui et al., (2015) |  | NA | Social Phobia | No | Yes ^4,5^ | Specific | Both | 12 |
| Etzelmueller et al., (in prep.) |  | Get.On Mood Enhancer | Depression | No | Yes ^6-11^ | Specific | Community | 6 |
| Gellatly et al., (2018) |  | Several [C] | Depression & Anxiety | No | Pilot RCT ^12-15^ [D] | Transdiagnostic | Clinical | NA |
| Hadjistavropoulos et al. (2014) | GAD [E] | NA | GAD [E] | No | NA | Specific | Both | 12 |
|  | Depression |  | Depression |  |  | Specific |  |  |
|  | Panic Disorder |  | Panic Disorder |  |  | Specific |  |  |
| Hadjistavropoulos et al. (2016) | Specialised Care | Wellbeing Course [F] | Depression & Anxiety | No | Yes ^16-18^ | Transdiagnostic | Both | 12 |
|  | Non-Specialised Care |  | Depression & Anxiety |  |  | Transdiagnostic | Both |  |
| Hedman et al. (2013) |  | NA [G] | Depression | No | Yes ^19,20^ | Specific | Both | 10 |
| Hedman et al. (2014) |  | NA [H] | Panic Disorder | No | Yes ^21,22^ | Specific | Both | 10 |
| Marks et al. (2003) | Phobia/  Panic | FearFighter | Phobia & Panic Disorder | No | Yes ^23-25^ | Specific | Both | 6 |
|  | Depression [I] | Cope | Depression / Anxiety | No | NA | Transdiagnostic |  | 6 |
|  | Anxiety/depression | Balance | Depression / GAD [E] | No | NA | Transdiagnostic |  | 3 |
|  | OCD [J] | BTSteps | OCD [J] | No | Yes ^26^ | Specific |  | 6 |
|  |  |  |  |  |  |  |  |  |
|  |  |  |  |  |  |  |  |  |
|  |  |  |  |  |  |  |  |  |
| Mathiasen et al. (2018) | Depression | NoDep® | Depression | No | NA | Specific | Community | 6+2 |
|  | Anxiety | FearFighter® | Panic Disorder, Agoraphobia, Social Phobia, Specific phobia, or GAD [E] | No | Yes ^23-25^ | Specific |  | 9 |
| Morrison et al. (2014) |  | MindBalance | Depression | No | NA | Specific | Clinical | 7 |
| Nordgreen et al. (2018) |  | NA | Panic Disorder | No | Yes ^27^ | Specific | Clinical | 9 |
| Nordgreen et al. (2018b) |  | NA | Social Anxiety Disorder | No | Yes ^28^ | Specific | Clinical | 9 |
| Ruwaard et al. (2012) | Depression | Web-CBT for Depression | Depression | No | Yes ^29-32^ | Specific | Community | NA |
|  | Panic Disorder | Web-CBT for Panic Disorder | Panic Disorder | No |  | Specific |  |  |
|  | PTSD [K] | Web-CBT for PTSD [K] | PTSD [K] | No |  | Specific |  |  |
| Shandley et al. (2008) | GP-guided | Panic Online | Panic Disorder | No | Yes ^33,34^ | Specific | Clinical | 6 |
|  | Therapist-guided |  | Panic Disorder | No |  | Specific | Community |  |
|  |  |  |  |  |  |  |  |  |
|  |  |  |  |  |  |  |  |  |
|  |  |  |  |  |  |  |  |  |
|  |  |  |  |  |  |  |  |  |
|  |  |  |  |  |  |  |  |  |
|  |  |  |  |  |  |  |  |  |
| (Titov et al., 2017) | Depression | The Wellbeing course [M] | Depression | No | Yes [M] | Specific | Community | 6-8 |
|  | Depression [L] | The Wellbeing Plus course [N] | Depression | No | Yes [N] | Specific |  |  |
|  | OCD [J] | OCD course [O] | OCD [J] | No | Yes [O] | Specific |  |  |
|  | PTSD [K] | PTSD course [P] | PTSD [K] | No | Yes [P] | Specific |  |  |
| (Yu et al., 2018) |  | Lantern | GAD [E] | No | NA | Specific | Clinical | 6 |

*Note*. The full references of included publications are available in Appendix D. [A] The evidence-base of the intervention was established within an RCT. [B] The naming of treatment components was inconsistent, ranging from “modules” to “lessons”, “sessions” and “core components”; [C] Living Life to the Fullest Interactive, SilverCloud Health, Beating the Blues; [D] Living Life to the Full Interactive ^12^, SilverCloud Health ^13^; Beating the Blues (mixed results) ^13,14^; [E] Generalised Anxiety Disorder; [F] Transdiagnostic iCBT "TD-ICBT"; [G] Revised version of the programme developed by Bergström and colleagues ^35^; [H] Based on the self-help program developed by Andersson and colleague ^19^; [I] Transdiagnostic treatment for depressed; [J] Obsessive compulsive disorder; [K] Post-traumatic stress disorder; [L] Depression treatment for older adults; [M] The Wellbeing course [the Wellbeing course for adults aged 18 years and over with symptoms of anxiety or depression Dear and colleagues ^16^ and Titov and colleagues ^1-3^; [N] The Wellbeing Plus course [the Wellbeing Plus course for older adults aged 60 years and over with symptoms of anxiety and depression ^36^; [O] OCD course [the obsessive-compulsive disorder (OCD) course for adults aged 18 and older with symptoms consistent with OCD ^37^; [P] PTSD course [and the post-traumatic stress disorder (PTSD) course for adults aged 18 years and over with symptoms consistent with PTSD ^38^

References.

1. Titov N, Andrews G, Choi I, Schwencke G, Mahoney A. Shyness 3: Randomized controlled trial of guided versus unguided Internet-based CBT for social phobia. Aust N Z J Psychiatry. 2008;42(12):1030-1040. doi:10.1080/00048670802512107

2. Titov N, Andrews G, Schwencke G. Shyness 2: Treating social phobia online: Replication and extension. Aust N Z J Psychiatry. 2008;42(7):595-605. doi:10.1080/00048670802119820

3. Titov N, Andrews G, Schwencke G, Drobny J, Einstein D. Shyness 1: Distance Treatment of Social Phobia over the Internet. Vol 42.; 2008.

4. Furmark T, Carlbring P, Hedman E, et al. Guided and unguided self-help for social anxiety disorder: Randomised controlled trial. Br J Psychiatry. 2009;195(5):440-447. doi:10.1192/bjp.bp.108.060996

5. Andersson G, Carlbring P, Holmström A, et al. Internet-based self-help with therapist feedback and in vivo group exposure for social phobia: A randomized controlled trial. J Consult Clin Psychol. 2006;74(4):677-686. doi:10.1037/0022-006X.74.4.677

6. Buntrock C, Berking M, Smit F, et al. Preventing depression in adults with subthreshold depression: Health-economic evaluation alongside a pragmatic randomized controlled trial of a web-based intervention. J Med Internet Res. 2017;19(1):e5. doi:10.2196/jmir.6587

7. Buntrock C, Ebert DD, Lehr D, et al. Effect of a Web-Based Guided Self-Help Intervention for Prevention of Major Depression in Adults With Subthreshold Depression. Obstet Gynecol Surv. 2016;71(9):526-527. doi:10.1097/01.ogx.0000490201.67580.e4

8. Nobis S, Ebert DD, Baumeister H, Snoek F, Riper H, Berking M. Efficacy of a Web-Based Intervention With Mobile Phone Support in Treating Depressive Symptoms in Adults With Type 1 and Type 2 Diabetes : A Randomized Controlled Trial. Diabetes Care. 2015;38(5):776-783. doi:10.2337/dc14-1728

9. Nobis S, Ebert DD, Lehr D, et al. Web-based intervention for depressive symptoms in adults with types 1 and 2 diabetes mellitus: A health economic evaluation. Br J Psychiatry. 2018;212(4):199-206. doi:10.1192/bjp.2018.10

10. Schlicker S, Weisel KK, Buntrock C, et al. Do Nonsuicidal Severely Depressed Individuals with Diabetes Profit from Internet-Based Guided Self-Help? Secondary Analyses of a Pragmatic Randomized Trial. J Diabetes Res. 2019;2019:2634094. doi:10.1155/2019/2634094

11. Ebert DD, Lehr D, Heber E, Riper H, Cuijpers P, Berking M. Internet- and mobile-based stress management for employees with adherence-focused guidance: Efficacy and mechanism of change. Scand J Work Environ Heal. 2016;42(5):382-394. doi:10.5271/sjweh.3573

12. McClay C-A, Collins K, Matthews L, et al. A community-based pilot randomised controlled study of life skills classes for individuals with low mood and depression. BMC Psychiatry. 2015;15(1):17. doi:10.1186/s12888-015-0384-2

13. Richards D, Timulak L, O’Brien E, et al. A randomized controlled trial of an internet-delivered treatment: Its potential as a low-intensity community intervention for adults with symptoms of depression. Behav Res Ther. 2015;75:20-31. doi:10.1016/j.brat.2015.10.005

14. Littlewood E, Duarte A, Hewitt C, et al. A randomised controlled trial of computerised cognitive behaviour therapy for the treatment of depression in primary care: The Randomised Evaluation of the Effectiveness and Acceptability of Computerised Therapy (REEACT) trial. Health Technol Assess (Rockv). 2015;19(101). doi:10.3310/hta191010

15. Proudfoot J, Goldberg D, Mann A, Everitt B, Marks I, Gray JA. Computerized, interactive, multimedia cognitive-behavioural program for anxiety and depression in general practice. Psychol Med. 2003;33(2):217-227. doi:10.1017/S0033291702007225

16. Dear BF, Staples LG, Terides MD, et al. Transdiagnostic versus disorder-specific and clinician-guided versus self-guided internet-delivered treatment for generalized anxiety disorder and comorbid disorders: A randomized controlled trial. J Anxiety Disord. 2015;36:63-77. doi:10.1016/j.janxdis.2015.09.003

17. Titov N, Dear BF, Johnston L, et al. Improving Adherence and Clinical Outcomes in Self-Guided Internet Treatment for Anxiety and Depression: Randomised Controlled Trial. PLoS One. 2013;8(7). doi:10.1371/journal.pone.0062873

18. Titov N, Dear BF, Staples LG, et al. Disorder-specific versus transdiagnostic and clinician-guided versus self-guided treatment for major depressive disorder and comorbid anxiety disorders: A randomized controlled trial. J Anxiety Disord. 2015;35:88-102. doi:10.1016/J.JANXDIS.2015.08.002

19. Andersson G, Bergström J, Holländare F, Carlbring P, Kaldo V, Ekselius L. Internet-based self-help for depression: randomised controlled trial. Br J Psychiatry. 2005;187(5):456-461. doi:10.1192/bjp.187.5.456

20. Vernmark K, Lenndin J, Bjärehed J, et al. Internet administered guided self-help versus individualized e-mail therapy: A randomized trial of two versions of CBT for major depression. Behav Res Ther. 2010;48(5):368-376. doi:10.1016/j.brat.2010.01.005

21. Carlbring P, Bohman S, Brunt S, et al. Remote treatment of panic disorder: A randomized trial of internet-based cognitive behavior therapy supplemented with telephone calls. Am J Psychiatry. 2006;163(12):2119-2125. doi:10.1176/ajp.2006.163.12.2119

22. Carlbring P, Westling BE, Ljungstrand P, Ekselius L, Andersson G. Treatment of panic disorder via the Internet: A randomized trial of a self-help program. Behav Ther. 2001;32(4):751-764. doi:10.1016/S0005-7894(01)80019-8

23. Marks IM, Kenwright M, McDonough M, Whittaker M, Mataix-Cols D. Saving clinicians’ time by delegating routine aspects of therapy to a computer: A randomized controlled trial in phobia/panic disorder. Psychol Med. 2004;34(1):9-17. doi:10.1017/S003329170300878X

24. Mathiasen K, Riper H, Ehlers LH, Valentin JB, Rosenberg NK. Internet-based CBT for social phobia and panic disorder in a specialised anxiety clinic in routine care: Results of a pilot randomised controlled trial. Internet Interv. 2016;4:92-98. doi:10.1016/j.invent.2016.03.001

25. Schneider AJ, Mataix-Cols D, Marks IM, Bachofen M. Internet-Guided Self-Help with or without Exposure Therapy for Phobic and Panic Disorders. Psychother Psychosom. 2005;74(3):154-164. doi:10.1159/000084000

26. Greist JH, Marks IM, Baer L, et al. Behavior therapy for obsessive-compulsive disorder guided by a computer or by a clinician compared with relaxation as a control. J Clin Psychiatry. 2002;63(2):138-145. doi:10.4088/JCP.v63n0209

27. Nordgreen T, Haug T, Öst LG, et al. Stepped Care Versus Direct Face-to-Face Cognitive Behavior Therapy for Social Anxiety Disorder and Panic Disorder: A Randomized Effectiveness Trial. Behav Ther. 2016;47(2):166-183. doi:10.1016/j.beth.2015.10.004

28. Nordgreen T, Standal B, Mannes H, et al. Guided self-help via internet for panic disorder: Dissemination across countries. Comput Human Behav. 2010;26(4):592-596. doi:10.1016/j.chb.2009.12.011

29. Knaevelsrud C, Maercker A. Internet-based treatment for PTSD reduces distress and facilitates the development of a strong therapeutic alliance: a randomized controlled clinical trial. BMC Psychiatry. 2007;7(1):13. doi:10.1186/1471-244X-7-13

30. Lange A, Rietdijk D, Hudcovicova M, van de Ven J-P, Schrieken B, Emmelkamp PMG. Interapy: A controlled randomized trial of the standardized treatment of posttraumatic stress through the internet. J Consult Clin Psychol. 2003;71(5):901-909. doi:10.1037/0022-006X.71.5.901

31. Ruwaard J, Schrieken B, Schrijver M, et al. Standardized Web-Based Cognitive Behavioural Therapy of Mild to Moderate Depression: A Randomized Controlled Trial with a Long-Term Follow-Up. Cogn Behav Ther. 2009;38(4):206-221. doi:10.1080/16506070802408086

32. Ruwaard J, Broeksteeg J, Schrieken B, Emmelkamp P, Lange A. Web-based therapist-assisted cognitive behavioral treatment of panic symptoms: A randomized controlled trial with a three-year follow-up. J Anxiety Disord. 2010;24(4):387-396. doi:10.1016/j.janxdis.2010.01.010

33. Klein B, Richards JC, Austin DW. Efficacy of internet therapy for panic disorder. J Behav Ther Exp Psychiatry. 2006;37(3):213-238. doi:10.1016/j.jbtep.2005.07.001

34. Richards JC, Klein B, Austin DW. Internet cognitive behavioural therapy for panic disorder: Does the inclusion of stress management information improve end-state functioning? Clin Psychol. 2006;10(1):2-15. doi:10.1080/13284200500378795

35. Bergström J, Andersson G, Ljótsson B, et al. Internet-versus group-administered cognitive behaviour therapy for panic disorder in a psychiatric setting: A randomised trial. BMC Psychiatry. 2010;10(1):54. doi:10.1186/1471-244X-10-54

36. Titov N, Fogliati VJ, Staples LG, et al. Treating anxiety and depression in older adults: randomised controlled trial comparing guided V. self-guided internet-delivered cognitive–behavioural therapy . BJPsych Open. 2016;2(1):50-58. doi:10.1192/bjpo.bp.115.002139

37. Wootton BM, Dear BF, Johnston L, Terides MD, Titov N. Remote treatment of obsessive-compulsive disorder: A randomized controlled trial. J Obsessive Compuls Relat Disord. 2013;2(4):375-384. doi:10.1016/j.jocrd.2013.07.002

38. Spence J, Titov N, Johnston L, Jones MP, Dear BF, Solley K. Internet-based trauma-focused cognitive behavioural therapy for PTSD with and without exposure components: A randomised controlled trial. J Affect Disord. 2014;162:73-80. doi:10.1016/j.jad.2014.03.009

**Appendix F** iCBT service-related characteristics – Part II

| Publication | Sub-Study | Guid. focus [1] | Guid. format [2] | Guid. moment [3] | Prof. training [4] | iCBT training [5] | Super-vision | Guid. time [6] | Contacts [7] | Manual [8] | Procedure in case of deteriora- tion/ crisis [9] |
| --- | --- | --- | --- | --- | --- | --- | --- | --- | --- | --- | --- |
| Aydos et al., (2009) |  | Content, Motivation | Email | Reaction [10] | Psychiatrist [11] | NA | Yes | 175 [12] | 9 | NA | NA |
| Alaoui et al., (2015) |  | Content, Motivation | Message System | Reaction [10] | Licenced Psychologist [13] | No | NA | 140 | 14.84 | No | Yes [14] |
| Etzelmueller et al., (in prep.) |  | Content, Motivation, Admin. [15] | Message System | Weekly | Psychologist [16] | Yes | Yes | NA | NA | Yes | Yes [17] |
| Gellatly et al., (2018) |  | Content, Admin. [15]; [18] | Tele- phone | NA [19] | Lived experience coordinators | NA | Yes | NA | NA [20] | NA | Yes [21] |
| Hadjistavropoulos et al. (2014) | GAD [22] | Content, Motivation | Email | Weekly | Other [23] | Yes | Yes | NA | 4.98 | NA | Yes [24] |
|  | Depression |  |  |  |  |  |  | NA | 4.98 |  |  |
|  | Panic Disorder |  |  |  |  |  |  | NA | 4.98 |  |  |
| Hadjistavropoulos et al. (2016) | Specialised Care | Content, Motivation, Admin. | Message System | Weekly | Psychologist [25] | Yes | Yes | NA | 8.87 | NA | NA |
|  | Non-Specialised Care |  |  |  | Psychologist [26] |  |  |  | 9.51 |  |  |
| Hedman et al. (2013) |  | Content | Message System | Reaction [10] | Registrar Psychologist | NA | NA | NA | 14.8 | NA | NA |
| Hedman et al. (2014) |  | Content | Message System | Reaction [10] | Registrar Psychologist | NA | NA | 149 | 14.14 | NA | NA |
|  |  |  |  |  |  |  |  |  |  |  |  |
| Marks et al. (2003) | Phobia/ Panic | Content | Tele-phone or Face-to-face | Bi-weekly | Nurse-therapist | NA | NA | 104 | NA | NA | Yes [29] |
|  | Depression [27] |  |  |  |  |  |  | 46 |  |  |  |
|  | Anxiety/  depression |  |  |  |  |  |  | 43 |  |  |  |
|  | OCD [28] |  |  |  |  |  |  | 81 |  |  |  |
| Mathiasen et al. (2018) | Depression | Content, Motivation | Message System | Weekly or Bi-weekly | Psychologist [30] | NA | Yes | NA | NA | NA | Yes [31] |
|  | Anxiety |  | Tele- phone |  |  |  |  |  |  |  |  |
| Morrison et al. (2014) |  | Content, Admin. | Message System | Weekly or Bi-weekly | Wellbeing Practitioner [32] | NA | NA | NA | NA | N/A | Yes [33] |
| Nordgreen et al. (2018) |  | Content | Message System | Weekly | Therapist | Yes | Yes | NA | NA | NA | No |
| Nordgreen et al. (2018b) |  | NA | Email | Weekly | Therapist | Yes | Yes | NA | NA | NA | No |
| Ruwaard et al. (2012) | Depression | Content, Motivation | Message System | NA | Junior Therapist [35] | Yes | Yes | 1200 [12] | NA | Yes | Yes [36] |
|  | Panic Disorder |  |  |  |  |  |  | NA |  |  |  |
|  | PTSD [34] |  |  |  |  |  |  | 570 [12] |  |  |  |
| Shandley et al. (2008) | GP-guided | NA | Face-to-face | Weekly | GP [38] | NA | NA | NA | 7.14 | NA | No |
|  | Therapist-guided |  | Email | [37] | Psychologist |  |  | 378.62 [39] | 15.29 |  |  |
| (Titov et al., 2017) | Depression | NA | Email | NA | Reg. Psych., Nurse, Counsellor [41] | Yes | Yes | 131 | 33 | Yes | Yes [42] |
|  | Depression [40] |  |  |  |  |  |  | 153 | 30 |  |  |
|  | OCD [28] |  |  |  |  |  |  | 183 | 37 |  |  |
|  | PTSD [34] |  |  |  |  |  |  | 194 | 34 |  |  |
| (Yu et al., 2018) |  | Content, Motivation | Message System | NA | Coach [43] | NA | Yes | NA | NA | NA | Yes [44] |

*Note*. Full references are available in Appendix D. [1] Guidance focus, if the guidance provided was mainly content-focussed, motivational-focussed and administrative-focussed; [2] guidance delivery format (synchronous vs. asynchronous, within the treatment platform vs. outside, e.g. by email); [3] Guidance moment, when was the guidance provided, as a reaction to an action of the participant [e.g. after the participant finished a session, as a reaction to a non-response], or planned in different intervals [e.g. weekly or bi-weekly]); [4] Guide’s professional training; trained as psychotherapist, psychiatrist, GP, psychologist, psychological registrar, Nurse, Coach [with lived experience]; [5] iCBT training; [6] Actual guidance intensity: reported guidance time in minutes; [7] Actual guidance intensity: reported number of contacts; [8] A guidance manual has been provided (yes/no); [9] Standardized procedure in case of symptom deterioration and crisis (yes/no) has been established; [10] Reaction after lesson/module; [11] or psychiatric registrar; [12] not included in data analysis; [13] (CBT) or Psychologist; [14] “… an automated monitoring system was built into the treatment software to alert therapists to process-related deviations (e.g., inactivity) or warning signs of suicidal ideation (i.e., when a patient scores 3 on MADRS–S Item 9). In such cases, patients (or a relative if unavailable) were generally contacted by tele- phone.”; [15] Administrative; [16] Licenced clinical Psychologist, Psychologist in training; [17] Standard operating procedures in case of crisis such as suicidal ideation included providing the client with detailed material on where to find help. In all cases, coaches would have to contact their supervisor; [18] Predominantly self-help; [19] Dependent on programme, 6-12 calls; [20] 4714 calls in total; [21] “Risk to self (i.e. self-harm or suicidal ideation) identified through discussions and in response to risk alerts sent by the program is monitored by coordinators who liaise regularly with clients’ GPs to inform them of risk, change in symptoms and discharge outcomes and act as a gateway into other services”; [22] Generalised Anxiety Disorder; [23] Community and students, social workers or psychologists; [24] “Therapists could also use clinical judgment to call patients at any point to offer additional support beyond emails (e.g., if patients reported suicidal thoughts)”; [25] Registrar Psychologist; social worker; Psychologist and social work graduate students; [26] Registrar Psychologist, social workers, Nurse, and counsellor; [27] Transdiagnostic treatment for depressed; [28] Obsessive compulsive disorder; [29] The computer faxed to the clinic weekly reports of patients’ tele- phone calls, their duration, the modules accessed and (for Cope patients) suicide risk – had this become high, which never happened, this would have been immediately faxed or phoned to the clinic”; [30] Licensed and/or clinical Psychologist; [31] “… the clinicians are notified of high scores on suicidal ideation and lack of progress. In both cases patients are contacted personally by phone and, in cases of suicidal risk, a standard procedure including risk assessment and possibly referral to an acute ward is instigated.”; [32] Trained psychology wellbeing practitioner; [33] “While there were measures in place to detect suicide risk…”; [34] Post-traumatic stress disorder; [35] With a degree in clinical Psychology; [36] "Psychiatric consultation was avail- able when needed.”; [37] Reaction after non-response; [38] trained in CBT; [39] 378.62 minutes (SD 264.43; n = 29) emailing participants throughout the 12-week treatment; [40] Depression treatment for older adults; [41] Registrar Psychologist, Nurse, counsellor (Mostly nationally registered psychologists); [42] “All patients were asked questions about suicidal thoughts, intentions and plans. As described in a recent report of the safety procedures used at the Clinic, a telephone-administered structured risk assessment was performed with all people who reported suicidal intent or plans, and safety plans were developed for all users to assist them to stay safe in the event of an increase in symptoms […]. People who were acutely suicidal were referred to local mental health services or emergency services, depending on the urgency of the situation”; Each week patients completed the GAD-7, PHQ-9 and single-item measures enquiring about personal safety and treatment satisfaction; [43] Master level coach with backgrounds in health and wellness coaching or mental health treatment; [44] Coaches review user messages daily for signs of high risk symptoms; provide crisis (e.g., county hotline, 911) or therapy referrals as appropriate; and notify Lantern staff and the study team of any risk issues and referrals”.
