# Supplementary material for: Effects of Internet-Based Cognitive Behavioral Therapy in Routine Care for Adults in Treatment for Depression and Anxiety: Systematic Review and Meta-Analysis
Source: J Med Internet Res. 2020 Aug 31;22(8):e18100. doi: 10.2196/18100 (PMC7490682; doi:10.2196/18100)
Supplement: Multimedia Appendix 6 [file jmir_v22i8e18100_app6.docx]

**Appendix G** Acceptability – Uptake and participant characteristics

| Publication |  | N screened [A] | N included [B] | N started [C] | Referral to included [D] | Referral to starter [E] | Included to starter [F] | Mean age (SD) | N females, (%) | Symptom Severity [G] | Modules completed [H] | Treatment duration  [I] |
| --- | --- | --- | --- | --- | --- | --- | --- | --- | --- | --- | --- | --- |
| Aydos et al., (2009) |  | 25 | 19 | 17 | 76.0 | 68.0 | 89.5 | 42.47 (9.54) | NA (71%) | 60.24 (9.27) [J] | 5.35 | 8 |
| Alaoui et al., (2015) |  | 654 | NA | 654 | NA | NA [K] | NA | 32.6 (10.2) | 360 (55%) | 70.77 (23.56) [L] | 8.12 | 13.5 |
| Etzelmueller et al., (in prep.) |  | NA | NA | 349 | NA | NA | NA | 41.68 (11.3) | 612 (72.2%) | 17.25 (2.83) [M] | 6.63 [N] | 6 |
| Gellatly et al., (2018) |  | 2054 | NA | 724 | NA | 35.3 | NA | 35.4 (11.6) | 447 (61.7%) | 11.9 (4.77) [O] | NA | 7 |
| Hadjistavropoulos et al. (2014) | GAD [P] | 379 | 221 | 212  [Q] | 58.3 | 55.9 | 95.9 | 38.71 (11.87) | 72 (64.3%) | 11.92 (5.27) [R] | 8.59 | 19.33 |
|  | Depression |  |  |  |  |  |  | 41.73 (13.27) | 62 (74.7%) | 14.75 (5.56) [S] | 8.42 | 18.19 |
|  | Panic Disorder |  |  |  |  |  |  | 39.38 (16.28) | 19 (73.1%) | 13.1 (6.45) [T] | 8.92 | 17.4 |
| Hadjistavropoulos et al. (2016) | Specialised Care | 792 | 679 | 458 | 85.7 | 57.8 | 67.5 | 38.6 (12.28) | 193 (74.2%) | 12.8 (5.78) [U] | NA | 14.64 |
|  | Non-Specialised Care |  |  |  |  |  |  | 39.5 (13.06) | 145 (73.2%) | 12 (5.38) [V] |  | 14.6 |
| Hedman et al. (2013) |  | 921 | NA | 570 | NA | 61.9 | NA | 37.3 (10.7) | 346  (NA) | 13.64 (5.18) [W] | 7.1 | 10-12 [X] |
| Hedman et al. (2014) |  | NA | NA | 1203 | NA | NA | NA | 37.9 (11.8) | 808 (67.2%) | 10.5 (4.9) [T] | 7.16 | 12 |
| Marks et al. (2003) | Phobia/Panic | 355 | 210 | 107 [AA] | 59.2 | 30.1 | 51.0 | 39 (12.0) | NA [AB] | 23.5 (11.5) [AC] | 5 | 9.57 |
|  | Depression [Y] |  |  |  |  |  |  |  |  | 27.4 (9.0) [AD] | NA | 9.29 |
|  | Anxiety/depression |  |  |  |  |  |  |  |  | 22.0 (10.0) [AE] | 2 | 5.71 |
|  | OCD [Z] |  |  |  |  |  |  |  |  | 23.2 (7.4) [AF] | NA | 9 |
| Mathiasen et al. (2018) | Depression | NA | NA | 60 | NA | NA | NA | 36.03 (10.97) | 47 (78.3%) | 15.47 (5.68) [S] | 5.37 | 12 |
|  | Anxiety | NA | NA | 143 | NA | NA | NA | 36.8 (13.550 | 94 (65.7%) | 10.42 (5.11) [R] | 5.94 |  |
| Morrison et al. (2014) |  | 4500 | 29 | 12 | 0.6 | 0.3 | 41.4 | NA [AG] | 11 | 14.25 (5.14) [AH] | 9.33 [AI] | 13.04 |
| Nordgreen et al. (2018) |  | NA |  | 114 | NA | NA | NA | 35.9 (11.77) | 41 (65.3%) | 45.89 (10.39) [AJ] | 6.2 | 12 |
| Nordgreen et al. (2018b) |  | NA | 169 | 145 | NA | NA | 85.8 | 29.8 (10.6) | 96 (56.8%) | 2.73 (2.73) [AK] | 5.3 | 9 |
| Ruwaard et al. (2012) | Depression | 3003 | 1500 | 609 [AM] | 50.0 | 20.3 | 40.6 | 40 (11) | NA | 24.2 (8.0) [AN] | NA | 22 |
|  | Panic Disorder |  |  |  |  |  |  | 37 (11) |  | 11.5 (5.8) [T] |  | 19 |
|  | PTSD [AL] |  |  |  |  |  |  | 38 (12) |  | 24.6 (7.3) [AO] |  | 8 |
| Shandley et al. (2008) | GP-guided | 53 | NA | 51 | NA | 96.2 | NA | 38.7 (10.9) | 43 | 16.05 (5.45) [AP] | NA | 12 |
|  | Therapist-guided | 43 | NA | 41 | NA | 95.4 | NA | 43.5 (12.4) | 33 | 14.62 (4.4) [AQ] |  | 12 |
| (Titov et al., 2017) | Depression | 33990 | NA | 6149  [AS] | NA | 18.1 | NA | 39.8 (13.3)  [L] | 4372  (71.1%)  [L] | 13.9 (6.0) [S] | NA | 8 |
|  | Depression [AR] |  |  |  |  |  |  |  |  | 12.7 (6.0) [S] |  |  |
|  | OCD [Z] |  |  |  |  |  |  |  |  | 24.0 (6.7) [AT] |  |  |
|  | PTSD [AL] |  |  |  |  |  |  |  |  | 62.5 (12.2) [AU] |  |  |
| (Yu et al., 2018) |  | NA | NA | 593 | NA | NA | NA | 40.4 (13.4) | 14 (22.2%) | 11.5 (4.8) [R] | NA | [AV] |

*Note.* The full references for the included publications are available in Appendix D. [A] Number of people referred to and/or screened for the service; [B] Number of eligible and/or included people; [C] Number of participants starting the treatment; [D] Proportion of included people based on the number of people screened; [E] Proportion of starters based on the number of people being screened; [F] Proportion of starters based on the number of people included; [G] Symptom Severity at baseline; [H] Number of completed modules; [I] Mean treatment duration in weeks; [J] on SIAS (Social Interaction Anxiety Scale ^1^); on SPS (Social Phobia Scale ^1^): 35.47 (17.86); [K] this study reports n = 615 participants reporting screening data, and n = 635 providing pre-treatment data, while including all starters (n = 654) into the analysis; [L] on LSAS-SR ( Liebowitz Social Anxiety Scale ^2^); [M] on PHQ8 ^3,4^; [N] Patients could access additional modules after following the intervention's main modules; [O] on PHQ9 ^3,4^; GAD7 (Generalised Anxiety Disorder 7-Item Scale ^5^): 11.3 (4.26); [P] Generalised Anxiety Disorder; [Q] In this study, of n = 212 starting the intervention, n = 107 started the GAD, n = 80 started the depression, and n = 25 started the panic disorder intervention; [R] on GAD7; [S] on PHQ9; [T] on PDSS-SR (Panic Disorder Severity Scale—Self Report ^6^); [U] on PHQ9; GAD7: 12.1 (5.36), PDSS-SR: 7.7 (6.83), SDS (Sheehan Disability Scale ^7^): 18 (7.92), SIAS: 13.9 (10.38); [V] on PHQ9; GAD7: 11.1 (5.06), PDSS-SR: 7.3 (6.13), SDS: 17.4 (7.82), SIAS: 12.3 (9.89); [W] on PHQ; MADSR-SR (Montgomery-Åsberg Depression Rating Scale– Self-Rated ^8^): 22.03 (6.55); [X] Mean of 11 in data set; [Y] Transdiagnostic treatment for depressed; [Z] Obsessive compulsive disorder; [AA] In Marks’ study ^9^: Of the n = 107 participants staring the intervention, n = 27 started the anxiety, n = 38 the depression, n = 33 the mixed and n = 9 the OCD treatment; [AB] n = 57 (of 108 participants), 53% (of 108 participants); [AC] on FQ(Anxiety/Depression); FQ-G ( Fear Questionnaire ^1^): 5.6 (1.7); FQ-T: 49 (27.1); [AD] on BDI (Beck Depression Inventory ^11^); HRSD ( Hamilton Depression Rating Scale ^12^): 16.8 (5.2); [AE] on BDI; BAI ( Beck Anxiety Inventory ^13^): 20.7 (11.9); [AF] on YBOCS (Yale– Brown Obsessive–Compulsive Scale ^14^); [AG] Values only for sub-groups and only frequencies; [AH] on PHQ-0; BDI: 24 (9.16); [AI] Adjusted for outliers; [AJ] on SIAS; SPS: 40.56 (15.14); [AK] on BSQ (Body Sensation Questionnaire ^15^); ACQ (Agoraphobic Cognitions Questionnaire ^15^): 2.47 (0.7), MI-A (The Mobility Inventory^16^): 2.55 (0.93), MI-WC: 1.98 (0.68); [AL] Post-traumatic stress disorder; [AM] For the main analysis, we excluded n = 470 participants from our analysis, as they received a burnout-related treatment. For this analysis on uptake, we included these participants to give a more accurate picture on how many participants started a treatment; [AN] on BDI; DASS (Depression Anxiety Stress Scales ^17^): 22.0 (9.1); [AO] on IES-Intrusion (Impact of Event Scale^18^); Avoidance: 23.4 (8.5); [AP] on PDSS-SR; ASP ( Anxiety Sensitivity Profile ^19^): 3.4 (1.42), DASS-Anxiety: 19.24 (9.8); [AQ] on PDSS-SR; ASP: 3.45 (1.31), DASS-Anxiety: 17.46 (10.1); [AR] Depression treatment for older adults; [AS] Of the n = 6149 starters, n = 5427 started the depression, n = 516 the "depression for elderly", n = 69 the OCD and n = 137 the PTSD treatment; [AT] on YBOCS; GAD7: 13.2 (5.3); [AU] on PCL-C ( Post-Traumatic Stress Disorder Checklist–Civilian Version ^20^); GAD7: 13.0 (5.1); [AV] Measurement period of eight weeks while the access to intervention was 2 years.

References

1. Mattick RP, Clarke JC. Development and validation of measures of social phobia scrutiny fear and social interaction anxiety. Behav Res Ther. 1998;36(4):455-470. doi:10.1016/S0005-7967(97)10031-6

2. Baker SL, Heinrichs N, Kim HJ, Hofmann SG. The Liebowitz social anxiety scale as a self-report instrument: A preliminary psychometric analysis. Behav Res Ther. 2002;40(6):701-715. doi:10.1016/S0005-7967(01)00060-2

3. Kroenke K, Spitzer RL. The PHQ-9: A New Depression Diagnostic and Severity Measure. Psychiatr Ann. 2002;32(9):509-515. doi:10.3928/0048-5713-20020901-06

4. Kroenke K, Spitzer RL, Williams JBW. The PHQ-9. J Gen Intern Med. 2001;16(9):606-613. doi:10.1046/j.1525-1497.2001.016009606.x

5. Spitzer RL, Kroenke K, Williams JBW, Löwe B. A brief measure for assessing generalized anxiety disorder: The GAD-7. Arch Intern Med. 2006;166(10):1092-1097. doi:10.1001/archinte.166.10.1092

6. Houck PR, Spiegel DA, Shear MK, Rucci P. Reliability of the self-report version of the panic disorder severity scale. Depress Anxiety. 2002;15(4):183-185. doi:10.1002/da.10049

7. Sheehan KH, Sheehan D V. Assessing treatment effects in clinical trials with the Discan metric of the Sheehan Disability Scale. Int Clin Psychopharmacol. 2008;23(2):70-83. doi:10.1097/YIC.0b013e3282f2b4d6

8. Svanborg P, Åsberg M. A new self-rating scale for depression and anxiety states based on the Comprehensive Psychopathological Rating Scale. Acta Psychiatr Scand. 1994;89(1):21-28. doi:10.1111/j.1600-0447.1994.tb01480.x

9. Marks IM, Mataix-Cols D, Kenwright M, Cameron R, Hirsch S, Gega L. Pragmatic evaluation of computer-aided self-help for anxiety and depression. Br J Psychiatry. 2003;183(01):57-65. doi:10.1192/bjp.183.1.57

10. Marks IM, Mathews AM. Brief standard self-rating for phobic patients. Behav Res Ther. 1979;17(3):263-267. doi:10.1016/0005-7967(79)90041-X

11. Beck AT, Ward CH, Mendelson M, Mock J, Erbaugh J. An Inventory for Measuring Depression. Arch Gen Psychiatry. 1961;4(6):561-571. doi:10.1001/archpsyc.1961.01710120031004

12. Hamilton M. A rating scale for depression. J Neurol Neurosurg Psychiatry. 1960;23(1):56-62. doi:10.1136/jnnp.23.1.56

13. Beck AT, Epstein N, Brown G, Steer RA. An Inventory for Measuring Clinical Anxiety: Psychometric Properties. J Consult Clin Psychol. 1988;56(6):893-897. doi:10.1037/0022-006X.56.6.893

14. Goodman WK, Price LH, Rasmussen SA, et al. The Yale-Brown Obsessive Compulsive Scale: I. Development, Use, and Reliability. Arch Gen Psychiatry. 1989;46(11):1006-1011. doi:10.1001/archpsyc.1989.01810110048007

15. Chambless DL, Caputo GC, Bright P, Gallagher R. Assessment of fear of fear in agoraphobics: The Body Sensations Questionnaire and the Agoraphobic Cognitions Questionnaire. J Consult Clin Psychol. 1984;52(6):1090-1097. doi:10.1037/0022-006X.52.6.1090

16. Chambless DL, Caputo GC, Jasin SE, Gracely EJ, Williams C. The Mobility Inventory for Agoraphobia. Behav Res Ther. 1985;23(1):35-44. doi:10.1016/0005-7967(85)90140-8

17. Nieuwenhuijsen K, De Boer AGEM, Verbeek JHAM, Blonk RWB, Van Dijk FJH. The Depression Anxiety Stress Scales (DASS): Detecting anxiety disorder and depression in employees absent from work because of mental health problems. Occup Environ Med. 2003;60(SUPPL. 1). doi:10.1136/oem.60.suppl_1.i77

18. Horowitz M, Wilner N, Alvarez W. Impact of event scale: A measure of subjective stress. Psychosom Med. 1979;41(3):209-218. doi:10.1097/00006842-197905000-00004

19. Taylor S, Cox BJ. Anxiety sensitivity: Multiple dimensions and hierarchic structure. Behav Res Ther. 1998;36(1):37-51. doi:10.1016/S0005-7967(97)00071-5

20. McDonald SD, Calhoun PS. The diagnostic accuracy of the PTSD Checklist: A critical review. Clin Psychol Rev. 2010;30(8):976-987. doi:10.1016/j.cpr.2010.06.012
