# Supplementary material for: Effects of Internet-Based Cognitive Behavioral Therapy in Routine Care for Adults in Treatment for Depression and Anxiety: Systematic Review and Meta-Analysis
Source: J Med Internet Res. 2020 Aug 31;22(8):e18100. doi: 10.2196/18100 (PMC7490682; doi:10.2196/18100)
Supplement: Multimedia Appendix 7 [file jmir_v22i8e18100_app7.docx]

**Appendix H** Acceptability - Participant satisfaction and negative effects

| Publication |  | Participant satisfaction - Questionnaire used | Satisfied or mostly satisfied | Summary (rated as) | Deterior- ation rate (%) | Adverse events reported | Report of specific subgroups at risk for symptom deterioration |
| --- | --- | --- | --- | --- | --- | --- | --- |
| Aydos et al., (2009) |  | CEQ [A] | 100% | Very high | NA | No | NA |
| Alaoui et al., (2015) |  | CSQ–8 [B] | - | High | NA | No | NA |
| Etzelmueller et al., (in prep.) |  | CSQ–8 [B] | 86% | High | 3.8 | Deterioration | NA |
| Gellatly et al., (2018) |  | - | - | - | 4.4 | Deterioration | NA |
| Hadjistavropoulos et al. (2014) | GAD [C] | Own [D] | - | Very high | NA | No | NA |
|  | Depression |  |  |  |  |  |  |
|  | Panic Disorder |  |  |  |  |  |  |
| Hadjistavropoulos et al. (2016) | Specialised Care | Own [E] | 94.80% | (Very) High | 1.0 | Deterioration | NA |
|  | Non-Specialised Care |  |  |  |  |  |  |
| Hedman et al. (2013) |  | CSQ–8 [B] | - | High | NA | No | NA |
| Hedman et al. (2014) |  | CSQ–8 [B] | - | High | NA | [F] | NA |
| Marks et al. (2003) | Phobia/Panic | Own [I] | - | High | NA | No | NA |
|  | Depression [G] |  |  |  |  |  |  |
|  | Anxiety/depression |  |  |  |  |  |  |
|  | OCD [H] |  |  |  |  |  |  |
| Mathiasen et al. (2018) | Depression | - | - | - | NA | No | NA |
|  | Anxiety |  |  |  |  |  |  |
| Morrison et al. (2014) |  | - | - | - | NA | No | NA |
| Nordgreen et al. (2018) |  | - | - | - | 2.6 | Deterioration | NA |
| Nordgreen et al. (2018b) |  | - | - | - | 16.6 | Deterioration | NA |
| Ruwaard et al. (2012) | Depression | Own [K] | - | High | 2 | Deterioration | NA |
|  | Panic Disorder |  |  |  |  |  |  |
|  | PTSD [J] |  |  |  |  |  |  |
| Shandley et al. (2008) | GP-guided |  | - | - | NA | No | NA |
|  | Therapist-guided |  |  |  |  |  |  |
| (Titov et al., 2017) | Depression | Own [M] | 7.6/95.5% | Very high | 2.2 | Deterioration | NA |
|  | Depression [L] |  |  |  | 1 |  |  |
|  | OCD [H] |  |  |  | 4.3 |  |  |
|  | PTSD [J] |  |  |  | 2.9 |  |  |
| (Yu et al., 2018) |  | - | - | - | NA | [N] | NA |

*Note*. Full references are available in Appendix D. [A] CEQ = “Credibility/ Expectancy Questionnaire”, CEQ [1,2]); [B] CSQ = “Client Satisfaction Questionnaire”, CSQ [3]; [C] Generalised Anxiety Disorder; [D] “How much did you like the treatment program?” and “How much did you enjoy communicating with your therapist?”. These two questions were rated on a 0 (not at all) to 7 (very much so) scale; [E] Consistent with past research on TD-ICBT [4], patients responded “yes” or “no” to indicate whether they would feel confident recommending the treatment to a friend and whether completing the course was worth their time; [F] “Another important feature of the treatment platform is that it automatically reports signs of potential risk for dropout or symptom deterioration. For example, the treating psychologists are notified of patients who have not reported any homework for more than a week or have scored >3 on the suicide ideation item (number nine) of the MADRS-S”; [G] Transdiagnostic treatment for depressed; [H] Obsessive compulsive disorder; [I] Satisfaction (at post-treatment) rated on four questions; [J] Post-traumatic stress disorder; [K] Patient satisfaction was assessed through a brief evaluation questionnaire, which was administered at post-test. Patients rated the contact with their therapists (on a 1–10 scale), and stated whether they perceived the treatment as effective, whether they had missed the face to face contact, and whether they would recommend the treatment to others (Yes/No/Don’t know)”; [L] Depression treatment for older adults; [M] Single-item measures enquiring about personal safety and treatment satisfaction; [N] “There were no adverse outcomes”.

References

1. Borkovec TD, Nau SD. Credibility of analogue therapy rationales. J Behav Ther Exp Psychiatry. 1972;3(4):257-260. doi:10.1016/0005-7916(72)90045-6

2. Devilly GJ, Borkovec TD. Psychometric properties of the credibility/expectancy questionnaire. J Behav Ther Exp Psychiatry. 2000;31(2):73-86. doi:10.1016/S0005-7916(00)00012-4

3. Nguyen TD, Attkisson CC, Stegner BL. Assessment of patient satisfaction: Development and refinement of a Service Evaluation Questionnaire. Eval Program Plann. 1983;6(3-4):299-313. doi:10.1016/0149-7189(83)90010-1

4. Titov N, Dear BF, Staples LG, et al. Disorder-specific versus transdiagnostic and clinician-guided versus self-guided treatment for major depressive disorder and comorbid anxiety disorders: A randomized controlled trial. J Anxiety Disord. 2015;35:88-102. doi:10.1016/J.JANXDIS.2015.08.002
