# Supplementary material for: Effects of Internet-Based Cognitive Behavioral Therapy in Routine Care for Adults in Treatment for Depression and Anxiety: Systematic Review and Meta-Analysis
Source: J Med Internet Res. 2020 Aug 31;22(8):e18100. doi: 10.2196/18100 (PMC7490682; doi:10.2196/18100)
Supplement: Multimedia Appendix 8 [file jmir_v22i8e18100_app8.docx]

**Appendix I** References for studies targeting guided iCBT interventions for the treatment of anxiety reporting on pre-post effect sizes.

Allen, A. R., Newby, J. M., Mackenzie, A., Smith, J., Boulton, M., Loughnan, S. A., & Andrews, G. (2016). Internet cognitive–behavioural treatment for panic disorder: randomised controlled trial and evidence of effectiveness in primary care. BJPsych Open, 2(2), 154–162. <https://doi.org/10.1192/bjpo.bp.115.001826>

Andersson, G., Waara, J., Jonsson, U., Malmaeus, F., Carlbring, P., & Öst, L.-G. (2013). Internet-Based Exposure Treatment Versus One-Session Exposure Treatment of Snake Phobia: A Randomized Controlled Trial. <https://doi.org/10.1080/16506073.2013.844202>

Andersson, G., Waara, J., Jonsson, U., Malmaeus, F., Carlbring, P., & Öst, L. G. (2009). Internet-based self-help versus one-session exposure in the treatment of spider phobia: A randomized controlled trial. Cognitive Behaviour Therapy, 38(2), 114–120. <https://doi.org/10.1080/16506070902931326>

Berger, T., Boettcher, J., & Caspar, F. (2014). Internet-based guided self-help for several anxiety disorders: A randomized controlled trial comparing a tailored with a standardized disorder-specific approach. Psychotherapy, 51(2), 207–219. <https://doi.org/10.1037/a0032527>

Berger, T., Caspar, F., Richardson, R., Kneubühler, B., Sutter, D., & Andersson, G. (2011). Internet-based treatment of social phobia: A randomized controlled trial comparing unguided with two types of guided self-help. Behaviour Research and Therapy, 49(3), 158–169. <https://doi.org/10.1016/j.brat.2010.12.007>

Berger, T., Hohl, E., & Caspar, F. (2009). Internet-based treatment for social phobia: A randomized controlled trial. Journal of Clinical Psychology, 65(10), 1021–1035. <https://doi.org/10.1002/jclp.20603>

Bergström, J., Andersson, G., Ljótsson, B., Rück, C., Andréewitch, S., Karlsson, A., … Lindefors, N. (2010). Internet-versus group-administered cognitive behaviour therapy for panic disorder in a psychiatric setting: A randomised trial. BMC Psychiatry, 10(1), 54. <https://doi.org/10.1186/1471-244X-10-54>

Carlbring, P., Bohman, S., Brunt, S., Buhrman, M., Westling, B. E., Ekselius, L., & Andersson, G. (2006). Remote treatment of panic disorder: A randomized trial of internet-based cognitive behavior therapy supplemented with telephone calls. American Journal of Psychiatry, 163(12), 2119–2125. <https://doi.org/10.1176/ajp.2006.163.12.2119>

Carlbring, P., Gunnarsdóttir, M., Hedensjö, L., Andersson, G., Ekselius, L., & Furmark, T. (2007). Treatment of social phobia: randomised trial of internet-delivered cognitive-behavioural therapy with telephone support. British Journal of Psychiatry, 190(2), 123–128. <https://doi.org/10.1192/bjp.bp.105.020107>

Carlbring, P., Maurin, L., Törngren, C., Linna, E., Eriksson, T., Sparthan, E., … Andersson, G. (2011). Individually-tailored, Internet-based treatment for anxiety disorders: A randomized controlled trial. Behaviour Research and Therapy, 49(1), 18–24. <https://doi.org/10.1016/j.brat.2010.10.002>

Furmark, T., Carlbring, P., Hedman, E., Sonnenstein, A., Clevberger, P., Bohman, B., … Andersson, G. (2009). Guided and unguided self-help for social anxiety disorder: Randomised controlled trial. British Journal of Psychiatry, 195(5), 440–447. <https://doi.org/10.1192/bjp.bp.108.060996>

Hedman, E., Andersson, G., Ljótsson, B., Andersson, E., Rück, C., Mörtberg, E., & Lindefors, N. (2011). Internet-based cognitive behavior therapy vs. cognitive behavioral group therapy for social anxiety disorder: A randomized controlled non-inferiority trial. PLoS ONE, 6(3). <https://doi.org/10.1371/journal.pone.0018001>

Hedman, E., El Alaoui, S., Lindefors, N., Andersson, E., Rück, C., Ghaderi, A., … Ljótsson, B. (2014). Clinical effectiveness and cost-effectiveness of Internet- vs. group-based cognitive behavior therapy for social anxiety disorder: 4-Year follow-up of a randomized trial. Behaviour Research and Therapy, 59, 20–29. <https://doi.org/10.1016/j.brat.2014.05.010>

Ivarsson, D., Blom, M., Hesser, H., Enderby, P., Nordberg, R., & Andersson, G. (2014). Guided internet-delivered cognitive behavior therapy for post-traumatic stress disorder: A randomized controlled trial. INVENT, 1, 33–40. <https://doi.org/10.1016/j.invent.2014.03.002>

Johnston, L., Titov, N., Andrews, G., Spence, J., & Dear, B. F. (2011). A RCT of a transdiagnostic internet-delivered treatment for three anxiety disorders: Examination of support roles and disorder-specific outcomes. PLoS ONE, 6(11). <https://doi.org/10.1371/journal.pone.0028079>

Jones, S. L., Hadjistavropoulos, H. D., & Soucy, J. N. (2016). A randomized controlled trial of guided internet-delivered cognitive behaviour therapy for older adults with generalized anxiety. Journal of Anxiety Disorders, 37, 1–9. <https://doi.org/10.1016/j.janxdis.2015.10.006>

Kok, R. N., Van Straten, A., Beekman, A. T. F., & Cuijpers, P. (2014). Short-term effectiveness of web-based guided self-help for phobic outpatients: Randomized controlled trial. Journal of Medical Internet Research, 16(9). <https://doi.org/10.2196/jmir.3429>

Nordgren, L. B., Hedman, E., Etienne, J., Bodin, J., Kadowaki, Å., Eriksson, S., … Carlbring, P. (2014). Effectiveness and cost-effectiveness of individually tailored Internet-delivered cognitive behavior therapy for anxiety disorders ina primary care population: A randomized controlled trial. Behaviour Research and Therapy, 59, 1–11. <https://doi.org/10.1016/j.brat.2014.05.007>

Oromendia, P., Orrego, J., Bonillo, A., & Molinuevo, B. (2016). Internet-based self-help treatment for panic disorder: a randomized controlled trial comparing mandatory versus optional complementary psychological support. Cognitive Behaviour Therapy, 45(4), 270–286. <https://doi.org/10.1080/16506073.2016.1163615>

Paxling, B., Almlöv, J., Dahlin, M., Carlbring, P., Breitholtz, E., Eriksson, T., & Andersson, G. (2011). Guided Internet-delivered cognitive behavior therapy for generalized anxiety disorder: A randomized controlled trial. Cognitive Behaviour Therapy, 40(3), 159–173. <https://doi.org/10.1080/16506073.2011.576699>

Robinson, E., Titov, N., Andrews, G., McIntyre, K., Schwencke, G., & Solley, K. (2010). Internet treatment for generlized anxiety disorder: A randomized controlled trial comparing clinician vs. technician assistance. PLoS ONE, 5(6). <https://doi.org/10.1371/journal.pone.0010942>

Silfvernagel, K., Carlbring, P., Kabo, J., Edström, S., Eriksson, J., Månson, L., & Andersson, G. (2012). Individually tailored internet-based treatment for young adults and adults with panic attacks: Randomized controlled trial. In Journal of Medical Internet Research (Vol. 14). <https://doi.org/10.2196/jmir.1853>

Spence, J., Titov, N., Dear, B. F., Johnston, L., Solley, K., Lorian, C., … Schwenke, G. (2011). Randomized controlled trial of Internet-delivered cognitive behavioral therapy for posttraumatic stress disorder. Depression and Anxiety, 28(7), 541–550. <https://doi.org/10.1002/da.20835>

Titov, N., Andrews, G., Choi, I., Schwencke, G., & Mahoney, A. (2008). Shyness 3: Randomized controlled trial of guided versus unguided Internet-based CBT for social phobia. Australian and New Zealand Journal of Psychiatry, 42(12), 1030–1040. <https://doi.org/10.1080/00048670802512107>

Titov, N., Andrews, G., Johnston, L., Robinson, E., & Spence, J. (2010). Transdiagnostic Internet treatment for anxiety disorders: A randomized controlled trial. Behaviour Research and Therapy, 48(9), 890–899. <https://doi.org/10.1016/j.brat.2010.05.014>

Titov, N., Dear, B. F., Schwencke, G., Andrews, G., Johnston, L., Craske, M. G., & McEvoy, P. (2011). Transdiagnostic internet treatment for anxiety and depression: A randomised controlled trial. Behaviour Research and Therapy, 49(8), 441–452. <https://doi.org/10.1016/j.brat.2011.03.007>

Tulbure, B. T., Szentagotai, A., David, O., Ștefan, S., Månsson, K. N. T., David, D., & Andersson, G. (2015). Internet-Delivered Cognitive-Behavioral Therapy for Social Anxiety Disorder in Romania: A Randomized Controlled Trial. <https://doi.org/10.1371/journal.pone.0123997>

Van Ballegooijen, W., Riper, H., Klein, B., Ebert, D. D., Kramer, J., Meulenbeek, P., & Cuijpers, P. (2013). An Internet-based guided self-help intervention for panic symptoms: Randomized controlled trial. Journal of Medical Internet Research, 15(7). <https://doi.org/10.2196/jmir.2362>

Wims, E., Titov, N., Andrews, G., & Choi, I. (2010). Clinician-assisted Internet-based treatment is effective for panic: A randomized controlled trial. Australian and New Zealand Journal of Psychiatry, 44(7), 599–607. <https://doi.org/10.3109/00048671003614171>

Wootton, B. M., Dear, B. F., Johnston, L., Terides, M. D., & Titov, N. (2013). Remote treatment of obsessive-compulsive disorder: A randomized controlled trial. Journal of Obsessive-Compulsive and Related Disorders, 2(4), 375–384. <https://doi.org/10.1016/j.jocrd.2013.07.002>
